# Spatiotemporal evaluation of water quality, metal pollution, and human health risks in a dredged Urban River, New Jersey, USA

**DOI:** 10.1007/s10653-025-02579-6

**Published:** 2025-06-25

**Authors:** Oluwafemi Soetan, Qingzhi Zhu, Huan Feng

**Affiliations:** 1 Department of Earth and Environmental Studies, Montclair State University, Montclair, NJ USA; 2 School of Marine and Atmospheric Sciences, Stony Brook University, Stony Brook, NY USA

**Keywords:** Remedial sediment dredging, Health risk assessment, Lower Passaic river, Water quality index, Toxic metals

## Abstract

Although the short-term effectiveness of remedial sediment dredging (RSD) on mitigating sediment contamination in the lower Passaic river (LPR), New Jersey, USA, was previously established in previous studies, dredging impacts on overlying water and consequential effects on aquatic life and human health were not investigated through model-based risk assessment methods. This study assessed the influence of RSD on metal pollution and toxicological risks upstream and downstream of River Mile-3.4, which was the subject of a 2012 RSD. Over the pre-, during-, and post-dredging periods, threats to aquatic life and humans were greater upstream at River Mile-4 (WQI_avg._ ≥ 50, PI_avg._ = 0.9) compared to downstream (River Miles-0 &1), where WQI_avg._ < 50 and PI_avg._ = 0.6 & 0.4, respectively. Monte Carlo probabilistic health risk assessment revealed significant probabilities of carcinogenic (≥ 98%) and non-carcinogenic health risks (2–50%) across all study areas and periods, with the highest risks recorded during and shortly after dredging, underscoring the associated toxicant exposure risks to humans from RSD. Significant (*p* < 0.05; 0.01) inter-correlations between metals were recorded pre-, during, and post-dredging, with observed negative correlation indicating concentration changes between the dredging and post-dredging periods. Principal component analysis of measured parameters explained two potential categories of pollution sources—(i) in-situ recontamination from dredging residuals and remobilized metals, and (ii) ongoing anthropogenic contributions from combined sewer outfalls and surface runoff. Recent pollution trends in the study areas underscore the effect of seasonality and anthropogenic influences on toxic metal pollution in the LPR and highlight the need for a robust and consolidated protective remedial action comprising engineered and natural measures, policy and engineering control of ongoing pollution sources, and institutional controls to limit human exposure.

**Graphical abstract:**

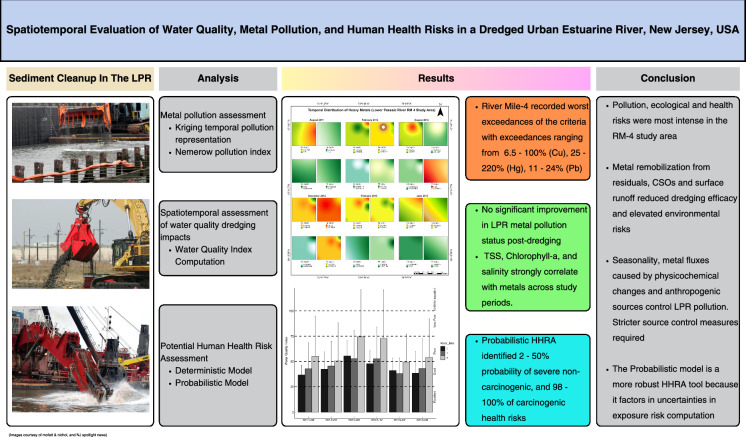

**Supplementary Information:**

The online version contains supplementary material available at 10.1007/s10653-025-02579-6.

## Introduction

The application of remedial sediment dredging (RSD) in contaminated sediment removal is widespread (Stauber et al., 2016). Consequently, many studies have examined its application from environmental, socioeconomic, and regulatory standpoints (Mohan et al., 2016; Soetan et al., 2023). Despite well-documented reports of RSD’s success in mitigating pollutants such as toxic metals (Guerra et al., 2009; Jiang et al., 2012; Mao et al., 2014; Smal et al., 2015), its capacity to effect sustained restoration and the ecological cost of its execution remain controversial, especially in boisterous systems that are constantly prone to contamination, such as urban rivers and estuaries. The geographic siting of these systems around human settlements denotes that they are at the receiving end of a deluge of anthropic stressors such as municipal sewage and debris, mining, construction, and maintenance dredging activities, agricultural and urban surface runoff, and industrial wastewater. The resultant effect of these pollution activities on aquatic systems is biodiversity loss and ecological imbalance due to chemical intoxication (Amoatey & Baawain, 2019; Kennish, 2017; Monte et al., 2023; Gandra et al., 2023; Li et al., 2023; Thippesh et al., 2023).

Toxic metals are high-potential contaminants of rivers and estuaries, which may be naturally available through rock weathering, erosion, volcanism, and atmospheric deposition, however, their major sources into aquatic systems are anthropogenic (Morais et al., 2012). Due to their degradation resistance, high bioaccumulative potential, and persistence, toxic metals are deleterious to aquatic ecosystems (Jiang et al., 2014). Their durability in the aquatic system stems from a peculiar transport and fate—they are scavenged by fine particles, organic matter, or Fe/Mn oxides, accumulated in sediments, and periodically dispersed into the water column or bioaccumulated in aquatic organisms based on the prevailing biological and physicochemical conditions (Gheorghe et al., 2017).

The predominant storage of toxic metals in sediments makes RSD a reasonable remedial option for pollution mitigation; however, its reputation as a potentially ecosystem-unfriendly mitigation approach is far-reaching for estuaries since they are critical biodiversity-rich zones that provide habitat to aquatic biota and perform numerous ecosystem services (García-Barcina et al., 2006). The application of RSD to toxic metal mitigation can aggravate the existing risks—Eggleston (2012) and Patmont et al. (2018) noted that the resulting residuals formed during sediment dredging, such as resuspended, metal-contaminated particulate matter and exposed layers of contaminated sediment bed, increase the ecological and health risks when metals are remobilized from them. Dredging also interferes with the physicochemical balance of aquatic systems by increasing turbidity and siltation, decreasing light penetration, creating a salinity gradient, and destroying habitats, thus negatively impacting fish biomass, density, and survival while also subjecting key marine species to starvation, smothering, shading, intoxication, and biomass decrease (Wenger Et Al., 2017).

The Lower Passaic River in Northern New Jersey, USA, continues to be associated with severe pollution and ecological degradation (Armstrong et al., 2005; Du et al., 2018; Ludwig & Iannuzzi, 2005; Onwueme & Feng, 2006). Despite previous remediation, public access to the river resources remains largely restricted due to enduring heightened pollution. A previous study addressed the efficacy of sediment dredging in mitigating metal pollution in the LPR and reported short-term effectiveness and long-term failures (Soetan et al., 2022). This resonates with similar reports in the literature about dredged polluted sites across the world (Monte et al., 2019; Rodrigues et al., 2020; Soetan et al., 2023). In this study, we seek to employ the use of established water quality (WQI) and health risk assessment (HHRA) models to understand the impacts of sediment dredging application to toxic metal mitigation in a local urbanized river estuary, on the local ecosystem and public health. This study is a continuation of our assessment of the impact of RSD on toxic metal pollution in the LPR and greatly enhances our current understanding of the environmental impacts of sediment dredging. We anticipate that this study will influence policy decisions for the LPR and other polluted rivers globally, and serve as a ‘lessons learned’ information to guide further remediation propositions for the river estuary.

## Materials and methods

### Study area

#### Geographical description

**Fig. 1:**
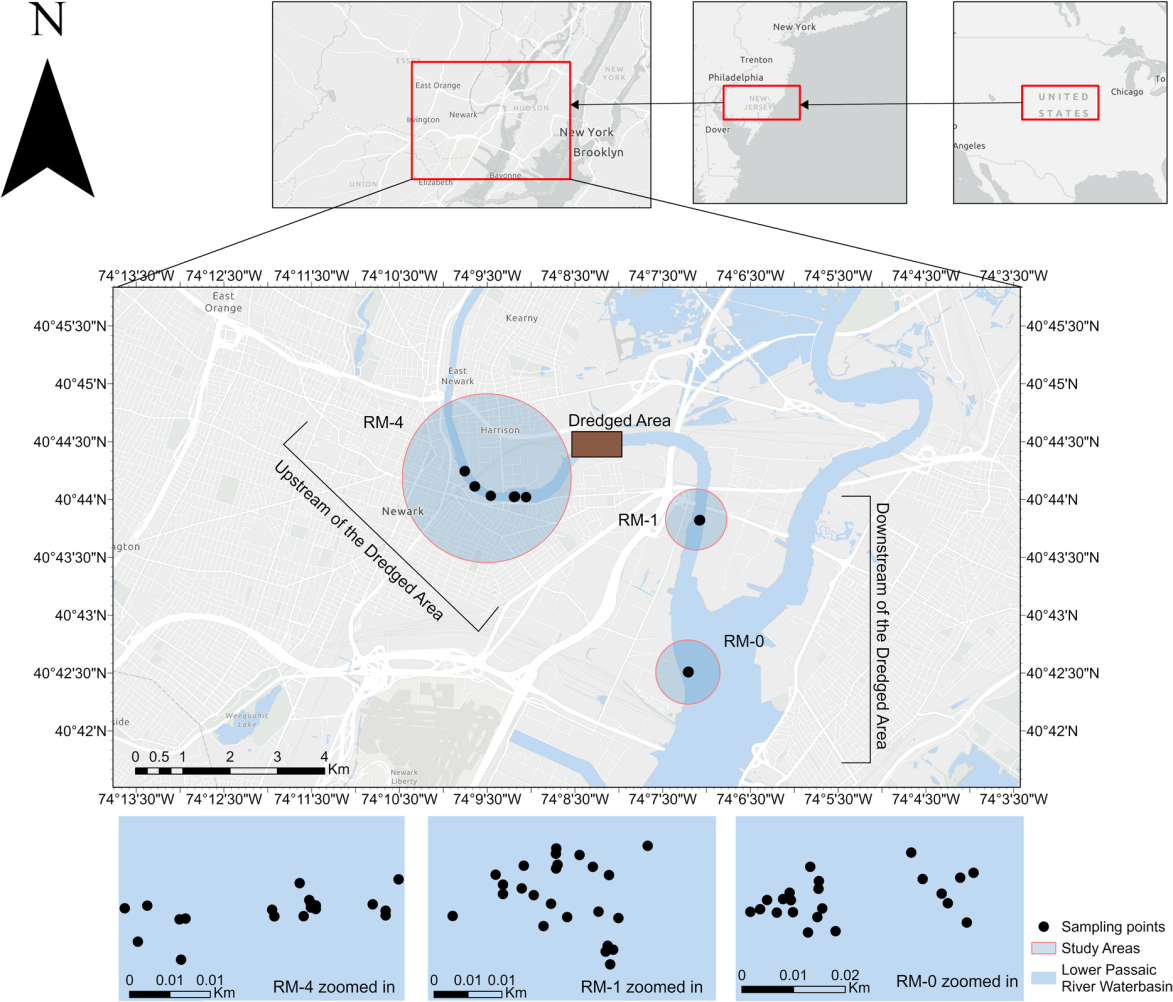
Map of the lower Passaic river study area showing the river miles 0, 1, and 4 study areas and the different surface water sampling locations from which data were collected for this study. Surface water sampling was conducted for pre-dredging periods of August 2011, February, during dredging periods of August, and December 2012, and in the post-dredging periods of February, and June 2013

The Passaic River basin is the third-largest drainage system in New Jersey, and it is exceeded only by the Delaware and Raritan River basins. The river rises from the Bernardsville mountains in Mendham Township. It covers an area of 2400 km^2^ of northern New Jersey, Rockland, and Orange counties of southern New York, draining several industrialized and dense watersheds across New Jersey and New York into the Newark Bay (Anderson & Faust, 1973). Our study area is located within the Lower 13 km of the Passaic River (74°7'—74°10' W and 40°42.9'—40°44.8' N), which is influenced by tides and forms a confluence with the lower Hackensack River at the mouth of Newark Bay. The specific study sites are downstream and upstream of an area previously dredged in the Tierra Phase One removal (River Mile 3.4). The study areas and sampling sites are shown in Fig. 1.

#### Pollution and remediation history

Bound by a vast urban landscape, the LPR has been historically subjected to decades-long pollution due to urbanization and industrialization (Soetan et al., 2022). The New Jersey Department of Environmental Protection’s 2004–2016 Land Use data shows that developed areas (residential, transportation, and industrial) make up the largest percentage of land use in the area (~65%) while green areas (pasture, shrubs, forest, cropland, grassland, wetlands) make up about 15–20% and have steadily decreased over the years (Nie, 2023). Critically, the LPR also receives a large proportion of raw and partially treated sewage from its surroundings, with 24% of New Jersey’s 212 active combined sewer outfalls discharging into the river (Soetan et al., 2022). Comprehensive environmental investigations by the US Environmental Protection Agency led to the discovery of elevated levels of toxic metals and organic pollutants, which led to the LPR’s characterization as an operable unit of the Diamond Alkali Superfund Site. Following a successful remediation pilot run in 2005, the first of a two-phase dredging operation in 2012 (Tierra Phase One) led to the removal of 30,500 m^3^ of contaminated sediment up to a 3.7 m depth from an identified contaminated section at River Mile 3.4 (RM-3.4) while the second phase projected to remove about 125,000 m^3^ remains uncompleted (Romagnoli & Bonkoski, 2012).

### Sample collection and analysis

Spatiotemporal data was extracted from the publicly available Lower Passaic River digital library at sharepoint.ourpassaic.org. Water column Cd, Cu, Hg, Pb, TSS, alkalinity, chlorophyll-a, salinity, and sulfate data were extracted for areas upstream (RM-4) and downstream (RM-1, RM-0) of the dredged area. The obtained data represented three dredging periods—pre-dredging (i.e., August 2011 and February 2012), during dredging (i.e., August and December 2012), and post-dredging (i.e., February and June 2013). The most recent metal data (June 2018 to March 2019) are extracted to evaluate longer-term environmental effects of sediment dredging on the study area.

The details of sample collection, analysis, and quality assurance and control have been described elsewhere in the project sampling and quality assurance reports (AECOM, 2012; Anchor QEA, 2019). In brief, 2L water samples were collected and preserved in ice at 0 - < 6°C and stored in the dark. Samples for metal analysis were stored and digested using HNO_3_, while BrCl was used for the preservation and oxidation of mercury samples. Pre-combusted glass fiber filters (GF/F) were used for all filtration purposes. The analytical methods used for toxic metal detection and analyses were according to EPA methods 6020-A and 6010-C for non-mercury metal analysis, and EPA method 1631 for mercury analysis. Sediment (TSS) concentration was determined by gravimetric analysis, while chlorophyll-a concentration was determined through spectrophotometric analysis. Sulfate concentration and alkalinity were measured using ion-chromatographic and titrimetric methods respectively. Method detection limits for alkalinity, Cd, chlorophyll-a, Cu, Hg, Pb, sulfate, and TSS were 3 mg L^−1^, 2 ng L^−1^, 0.5 µg L^−1^, 0.02 µg L^−1^, 0.15 ng L^−1^, 0.02 µg L^−1^, 3 mg L^−1^ and 2 mg L^−1^ respectively while the precision of all analyte data analysis was verified by the relative percent difference (RPD) (Eq. (1)).

1 $$RPD = \frac{{D_{1} - D_{2} }}{{\left( {\frac{{D_{1} + D_{2} }}{2}} \right)}} \times 100$$

where D_1_ and D_2_ represent the first and second (duplicate) sample values, respectively. RPD for all the analytes was within ≤ 20% and thus met the standards set by the method quality control measurement performance criteria (AECOM, 2012).

### Environmental risk assessment

The water quality index (WQI) and the human health risk assessment (HHRA) models are some of the most prominent models and indicators used to conduct environmental risk assessment of aquatic systems (Hoang et al., 2021). The WQI is a water quality assessment tool that incorporates several physicochemical properties (pH, dissolved oxygen, turbidity, temperature, nutrient concentration, solids concentration, metal concentrations, biochemical oxygen demand, and fecal coliform) to assess a water body’s viability and its ability to support life (Akter et al., 2016). Some of the several WQI models that have been developed globally include Horton WQI, NSF-WQI, SRDD-WQI, BCWQI, and CCME-WQI (Uddin et al., 2021). The HHRA model is a useful tool for measuring various populations’ carcinogenic and non-carcinogenic risk potential resulting from interaction with a water body’s resources (Goswami & Kalamdhad, 2022; Hashempour-baltork et al., 2023; Ogarekpe et al., 2023). The HHRA can be computed using two distinct approaches—the traditional, deterministic method and the probabilistic method. However, the probabilistic HHRA method, which simulates uncertainty through statistics and random sampling, is more robust as it accounts for the variations in parameters such as consumption rates, exposure frequencies, and body weight (Guo et al., 2023).

#### Water quality index

The water quality index (WQI) is a globally accepted metric for the determination of water quality and integrity within a defined study area (Howladar et al., 2021). The WQI procedure originally proposed by Brown et al. (1970) was used to assess LPR water quality due to its parameter flexibility.

2 $${\text{WQI}} = { }\frac{{\sum {\text{Q}}_{{\text{n}}} { } \times {\text{W}}_{{\text{i}}} }}{{\sum {\text{W}}_{{\text{i}}} }}$$

3 $${\text{Q}}_{{\text{n}}} = { }\frac{{{\text{C}}_{{\text{i}}} }}{{{\text{C}}_{{\text{s}}} }}$$

4 $$W_{i} = \frac{W}{\sum W}$$

Q_n_ and W_i_ are the quality value and unit weight of parameter i, respectively. C_i,_ and C_s_ are the measured and criterion values of parameter i, respectively, and W is the weight factor of parameter i. As a reference, WQI ≤ 25, 50, 75, and 100 denote excellent, good, poor, and very poor water quality, respectively, while WQI > 100 denotes that the water is unfit for aquatic life (Akoteyon et al., 2011). The parameters used for WQI calculation are presented in Table S1.

#### Toxic metal pollution assessment

The pollution index (PI) by Nemerow (1991) is a useful tool for investigating pollution contributions of individual elements. It was used for a comprehensive assessment of toxic metal pollution levels in the LPR.

5 $$C_{f} = \frac{{C_{n} }}{{S_{n} }}$$

6 $$PI = \sqrt {\frac{{C_{fmax}^{2} + C_{fave}^{2} }}{2}}$$

where C_n_ and S_n_ are the measured and the criteria values of the toxic element. C_fmax_ and C_fave_ are the maximum and mean calculated contamination factors of the toxic element. PI values ≤ 0.59, 0.74, 1, and 3.5 indicate no, very slight, slight, and moderate water pollution in that order, while PI > 3.5 represents severe pollution (Su et al., 2022a, b).

### Human health risk assessment (HHRA)

In this study, the HHRA was based on three exposure routes—dermal contact, accidental ingestion, and fish ingestion. The total carcinogenic (TCR) and non-carcinogenic (HI) risks are cumulatively computed from individual target cancer risk values (T_a_CR_n_) and the individual target hazard quotient (T_a_HQ_n_) values of each toxic metal studied (Howladar et al., 2021).

7 $${\text{TCR}} = { }\sum \left( {{\text{T}}_{{\text{a}}} {\text{CR}}} \right)_{{\text{n}}} = \sum {\text{CDI }} \times {\text{CSF}}$$

8 $${\text{HI}} = { }\sum \left( {{\text{T}}_{{\text{a}}} {\text{HQ}}} \right)_{{\text{n}}} = \sum \frac{{{\text{CDI}}}}{{{\text{Rf}}_{{\text{d}}} }}$$

where CDI is the chronic daily intake of the trace metal, CSF is the cancer slope factor, and Rf_d_ is the reference dose. The chronic daily intake is determined based on the route of exposure.

9 $${\text{CDI}}_{{{\text{dermal}}}} \left( {{\text{mg kg}}^{ - 1} {\text{d}}^{ - 1} } \right) = { }\frac{{{\text{SA }} \times {\text{ PC }} \times {\text{ CF }} \times {\text{ C}}_{{\text{n}}} \times {\text{ ET}}_{{{\text{sw}}}} { } \times {\text{ EF}}_{{{\text{sw}}}} { } \times {\text{ ED}}_{{{\text{sw}}}} }}{{{\text{BW }} \times {\text{ AT }} \times { }1000}}$$

10 $${\text{CDI}}_{{{\text{AI}}}} \left( {{\text{mg kg}}^{ - 1} {\text{d}}^{ - 1} } \right) = { }\frac{{{\text{R }} \times {\text{ C}}_{{\text{n}}} { } \times {\text{ ET}}_{{{\text{sw}}}} { } \times {\text{ EF}}_{{{\text{sw}}}} { } \times {\text{ ED}}_{{{\text{sw}}}} }}{{{\text{BW }} \times {\text{ AT}} \times { }1000}}$$

11 $${\text{CDI}}_{{{\text{FI}}}} \left( {{\text{mg kg}}^{ - 1} {\text{d}}^{ - 1} } \right) = { }\frac{{{\text{U }} \times {\text{ C}}_{{{\text{fish}}}} { } \times {\text{ EF}}_{{{\text{FI}}}} { } \times {\text{ ED}}_{{{\text{FI}}}} }}{{{\text{BW }} \times {\text{ AT }} \times { }1000}}$$

12 $$C_{{{\text{fish}}}} \left( {\mu g {\text{kg}}^{ - 1} } \right) = C_{n} \times BCF$$

where CDI_AI_ and CDI_FI_ are the chronic daily intakes from accidental water ingestion during swimming and from fish ingestion (Shen et al., 2019). The parameters used in the calculation of the carcinogenic and non-carcinogenic health effects are presented in Tables S1and S2. Non-carcinogenic health risks are negligible at HI ≤ 1 and significant at HI > 1. Carcinogenic health risks are negligible at TCR ≤ 10^-6^ but are significantly serious at TCR > 10^−4^ (Alipour et al., 2014).

### Statistical analysis

Comprehensive details of the statistical analysis of data are presented in the supplementary information. In brief, all statistical analyses, including homogeneity and normality tests, analysis of variance and significance, test for sampling adequacy and sphericity, correlation and principal component analyses, were executed with the R software. Monte Carlo probabilistic HHRA simulation and uncertainty analysis were executed in the Oracle Crystal Ball v11.1.3.0 software, while the spatiotemporal changes in toxic metal concentrations were represented using the kriging interpolation method on ArcGIS Pro v3.0.0.

## Results

### Spatiotemporal variation in trace metal and physicochemical variable concentrations

The statistical summary of trace metals and physicochemical data is shown in Table 1.

**Table 1:** Statistical data summary of trace metals and physicochemical properties of water based on LPR water column monitoring data collected from 2011 to 2013 before, during and after remedial sediment dredging

Parameters	Unit	Stat	RM 0						RM 1					
			08-2011	02-2012	08-2012	12-2012	02-2013	06-2013	08-2011	02-2012	08-2012	12-2012	02-2013	06-2013
Alkalinity	mg L^−1^	Mean	80.5	100	140	103	92	77	74	97	139	108	87	66
		SD	7.0	3.3	1.7	3.3	10.4	13.4	14.6	3.2	2.8	6.0	13.7	16.9
		Max	88	105	142	107	103	92	89	100	141	121	100	91.2
		Min	69	94	138	98	72	57	51	91	135	103	65	49
Cd	ng L^−1^	Mean	41	49	69	76	48	32	40	55	69	76	44	31
		SD	13	7	5	8	8	15	23	17	15	21	15	17
		Max	58	54	73	83	60	52	85	91	97	106	57	69
		Min	2	34	57	63	35	6	15	37	43	4	2	18
Chlorophyll-a	µg L^-1^	Mean	2.3	2.8	2.1	1.6	4.0	1.9	3.6	4.0	3.3	2.7	4.0	2.2
		SD	1.3	0.7	1.0	0.7	1.0	0.7	1.2	1.9	0.9	1.5	1.3	0.6
		Max	3.9	3.8	4.1	3.3	5.6	3.0	6.1	7.8	5.2	5.4	6.2	2.8
		Min	0.7	1.9	1.0	1.1	2.7	1.0	2.0	2.4	2.4	1.0	2.3	1.2
Cu	µg L^−1^	Mean	2	1.4	3.5	1.7	1.8	2.1	2.5	1.9	2.2	2.0	1.6	2.4
		SD	0.1	0.1	1.1	0.3	0.5	0.9	0.8	0.9	0.7	0.3	0.3	1.0
		Max	2.1	1.6	5.1	2.2	2.5	3.9	4.2	4.1	3.8	2.5	1.9	4.2
		Min	1.8	1.3	2.1	1.3	1.3	1.3	1.7	1.4	1.7	1.6	1.1	1.4
Hg	ng L^−1^	Mean	3.4	1.6	2.1	3.4	3	7.5	6.5	5.3	6.1	7.3	3.4	9.4
		SD	1.4	1.6	1.8	4.1	1.9	8.8	7.7	4.7	12.5	12.8	4.2	10.6
		Max	5.5	5.2	4.6	11.3	6.5	27.3	24.5	13.6	36.9	38.1	12.6	26.2
		Min	0.8	0.3	0.3	0.4	0.7	0.7	1.0	0.5	0.5	1.0	0.5	1.2
Pb	µg L^−1^	Mean	0.3	0.2	0.3	0.4	0.6	0.6	1.1	1.3	0.9	0.5	0.3	1.2
		SD	0.1	0.1	0.1	0.3	0.4	0.7	3.3	4.1	2.7	1.3	0.9	3.4
		Max	0.5	0.4	0.5	1.0	1.1	2.1	3.3	4.1	2.7	1.3	0.9	3.4
		Min	0.1	0.1	0.1	0.1	0.1	0.1	0.1	0.1	0.1	0.2	0.1	0.1
Salinity	g Kg^−1^	Mean	12.1	17.6	21.8	20.5	18.7	9.7	8.7	14.2	18.4	15.0	14.7	5.3
		SD	5.1	4.4	3.5	4.1	6.1	5.8	5.9	5.0	4.4	5.6	7.6	6.1
		Max	18.3	21.0	24.3	23.5	24.9	17.9	17.0	20.1	23.2	22.0	24.2	16.4
		Min	4.8	9.0	13.9	11.2	9.2	2.6	1.0	5.0	9.7	6.4	4.2	0.2
Sulfate	mg L^−1^	Mean	992	1153	1420	1584	1178	670	623	890	1203	1285	970	363
		SD	409	289	195	270	447	401	370	339	298	468	484	411
		Max	1570	1380	1600	1790	1670	1220	1130	1320	1520	1720	1560	1110
		Min	337	600	1020	960	480	188	70	310	600	500	250	10.4
TSS	mg L^−1^	Mean	14.8	26.3	19.9	23.0	14.5	15.9	23.2	23.7	22.9	29.6	16.8	26.7
		SD	5.3	24.9	13.9	7.9	3.9	5.8	16.3	19.2	19.2	27.7	6.8	15.9
		Max	24.7	87	49.4	39	19.4	29.2	61.9	70.8	69.3	96.7	25.3	60.8
		Min	8.2	13.2	9.4	15.6	10.5	11.3	10.3	12.1	9.1	12.9	7.7	9.7

Parameters	Unit	Stat	RM 4						Estuarine aquatic life criteria	References
			08-2011	02-2012	08-2012	12-2012	02-2013	06-2013		
Alkalinity	mg L^−1^	Mean	60	91	139	102	62	54	120	USEPA, (2006a)
		SD	11.6	4.3	1.6	6.4	10.0	7.3		
		Max	78.3	97.5	141	109	77.7	64.9		
		Min	49	86	136	91	51	47		
Cd	ng L^−1^	Mean	22	50	48	80	37	25	8800	USEPA, (2001)
		SD	4	25	16	17	14	14		
		Max	29	104	67	107	62	61		
		Min	17	33	25	64	24	2		
Chlorophyll-a	µg L^−1^	Mean	7.4	6.9	10.8	3.5	7.8	3.7	20	NJDEP, (2020)
		SD	3.2	2.8	11.6	1.2	2.2	1.6		
		Max	14.6	11.6	31.3	4.9	10.6	6.2		
		Min	3.8	3.3	1.9	1.8	4.8	1.8		
Cu	µg L^−1^	Mean	2.7	2.5	3.1	3.6	2.8	2.9	3.1	NJDEP, (2018)
		SD	0.8	1.0	1.3	2.0	0.7	0.8		
		Max	3.8	4.5	5.9	6.2	3.9	4.8		
		Min	1.6	1.4	2.2	1.5	1.7	2.3		
Hg	ng L^−1^	Mean	5.6	3.7	4.8	20.9	8	8.1	16	CCME, (2010)
		SD	5.4	4.7	3.5	25.5	6.7	11.7		
		Max	17.3	13.3	10.8	57.9	20.3	36.4		
		Min	0.7	0.3	0.8	0.5	1.1	1.7		
Pb	µg L^−1^	Mean	0.6	1.3	7.3	2.8	1.2	1.3	5.603	USEPA, (1984)
		SD	0.5	1.3	5.7	2.5	0.9	1.2		
		Max	1.6	4.2	21.0	5.9	2.6	4.0		
		Min	0.2	0.1	3.2	0.1	0.2	0.4		
Salinity	g Kg^−1^	Mean	4.3	6.8	12.8	9	1.8	2.2	10	USEPA, (2006b)
		SD	5.3	4.5	6.5	4.9	3.3	5.7		
		Max	11.3	12.6	19.1	16.3	9.9	16.3		
		Min	0.2	2.5	2.8	4.3	0.3	0.1		
Sulfate	mg L^−1^	Mean	140	520	719	730	186	10	2000	Iowa Department of Natural Resources, (2009)
		SD	191	306	359	375	288	1.8		
		Max	530	950	1180	1360	700	12.5		
		Min	11.4	140	202	350	13.7	7.9		
TSS	mg L^−1^	Mean	62.1	40.4	34.1	36.1	41.1	56.8	30	Qian et al., (2007)
		SD	57.8	48.4	36.5	15.1	28.0	43.2		
		Max	178	156	123	61.6	102	126		
		Min	14.1	12.7	10.5	18.4	8.7	12.6		

**Fig. 2:**
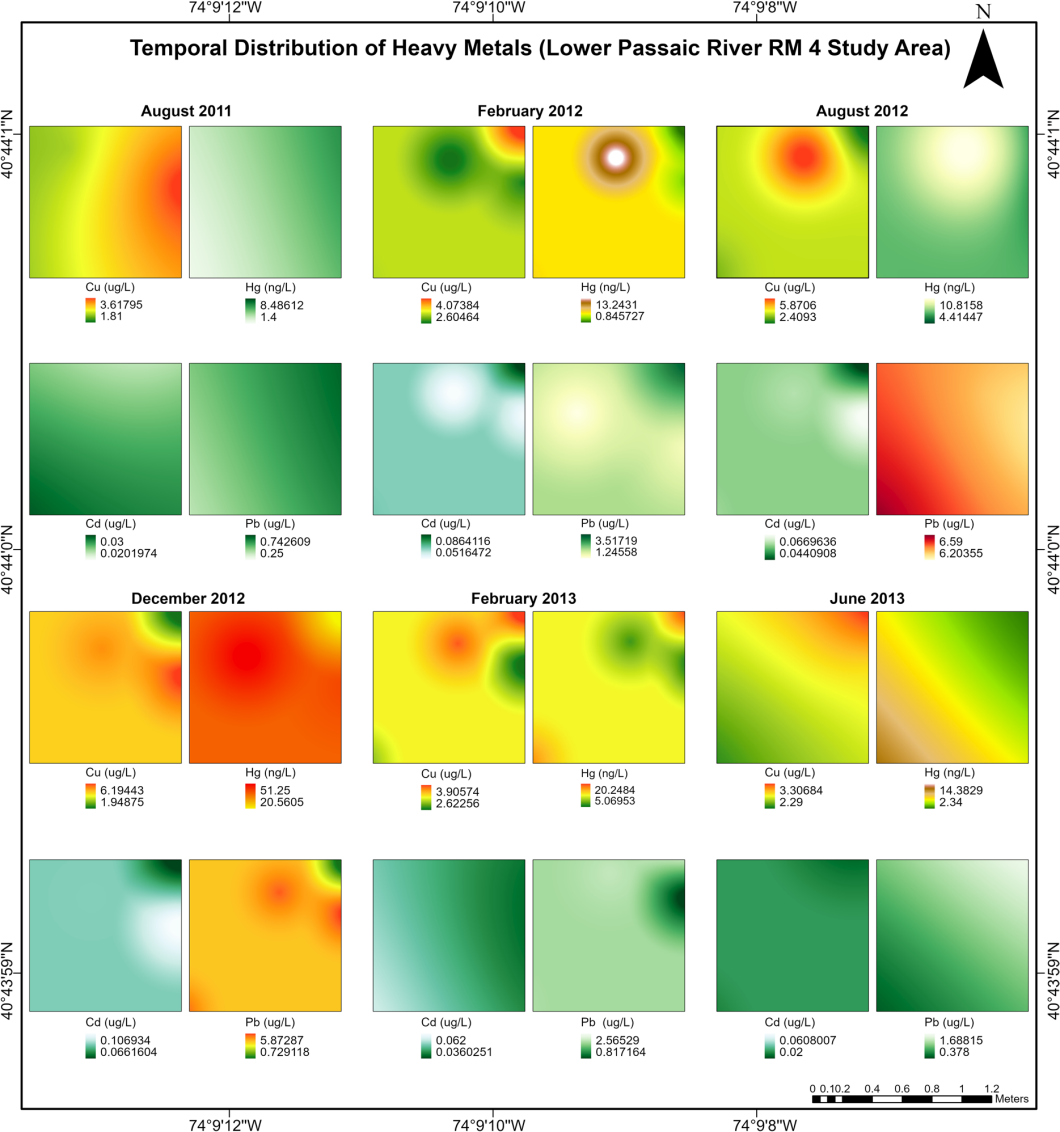
Changes recorded in water column toxic metal concentrations in the RM-4 study area from the pre-dredging period (August 2011) to six months post-dredging (June 2013)—Cu maximum concentrations were consistently above the recommended aquatic life criteria (RALC) while Cd levels were consistently below the RALC across the periods investigated. Cu, Hg and Pb recorded ‘above-RALC’ maximum concentrations in water column during dredging periods (August and December 2012)

Metal concentrations in RMs 0, 1, and 4 respectively ranged from 10–80 ng L^−1^, 20–110 ng L^−1^ and 20–110 ng L^−1^ for Cd, 1.27–5.09 µg L^−1^, 1.11–4.19 µg L^−1^ and 1.36–6.20 µg L^−1^for Cu, 0.26–27.30 ng L^−1^, 0.48–38.10 ng L^−1^, 0.29–57.90 ng L^−1^ for Hg, 0.07–2.14 µg L^−1^, 0.08–4.10 µg L^−1^, 0.11–21 µg L^−1^ for Pb (Fig. S1). All the metals recorded criteria exceedances in one or more periods and study areas except Cd. The toxic metal distribution indicated in Fig. 2 shows that Cu maximum recorded values were consistently above criteria across all study periods in the RM-4 study area, with significantly elevated (*p* < 0.05) levels of Hg and Pb recorded during dredging (August and December 2012). Post-dredging (June 2013) metal concentrations were slightly higher than pre-dredging levels. Total suspended solids (TSS) and chlorophyll-a levels were significantly higher (*p* < 0.05) in RM-4 before, during, and after dredging. Alkalinity, salinity, and sulfate levels were significantly higher (*p* < 0.05) for all study areas during dredging.

### Water quality assessment

**Fig. 3:**
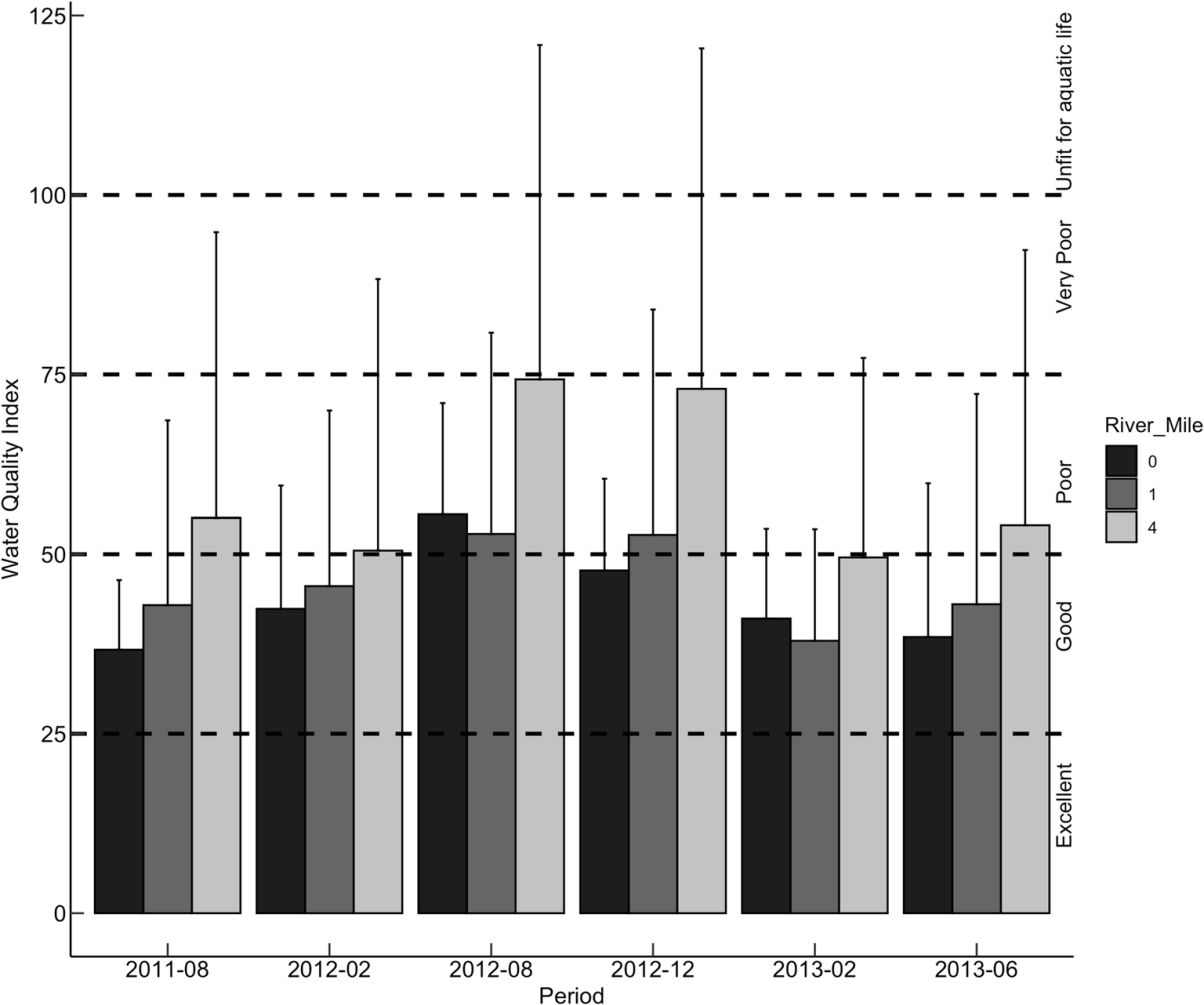
Water quality distribution at the three study areas (RMs −0, 1, and 4) during the different periods of study—Water quality ranged from “Poor” to “unfit for use” in the RM-4 study area, and ranged from “good” to “very poor” in the RM-1 and RM- 4 study areas. None of the study areas recorded “excellent” water quality

Average WQI values ranged from good to poor across all study areas 37–56 (RM-0), 38–53 in (RM-1), and 50–74 (RM-4). The poorest levels of water quality were recorded across all study areas during dredging (Fig. 3). Across most of the periods of study, water quality was consistently poor in RM-4, with at least one sampling location deemed unfit for aquatic life with a WQI of 121 during the dredging period. Water quality was good in RMs-0 and 1 before and after dredging; however, poor water quality was recorded in both areas during dredging. There was no significant difference in water quality between pre-dredging (August 2011) and six months post-dredging (June 2013).

### Pollution assessment

The Nemerow pollution index (PI) computation for individual metals is presented in Fig. S2. Based on the PI classification, Cd had no pollution effect across all study areas (PI_Cd_ < 0.1) while Cu, Hg, and Pb pollution effects ranged from very slight to moderate pollution across all study areas (PI_Cu_: 0.5–1.6, PI_Hg_: 0.2–2.7, PI_Pb_: 0.06–2.81). Based on pollution intensity, the RM-4 study area was the most polluted, predominantly in August 2011, August 2012, December 2012, and June 2013.

### Multivariate analysis

**Fig. 4:**
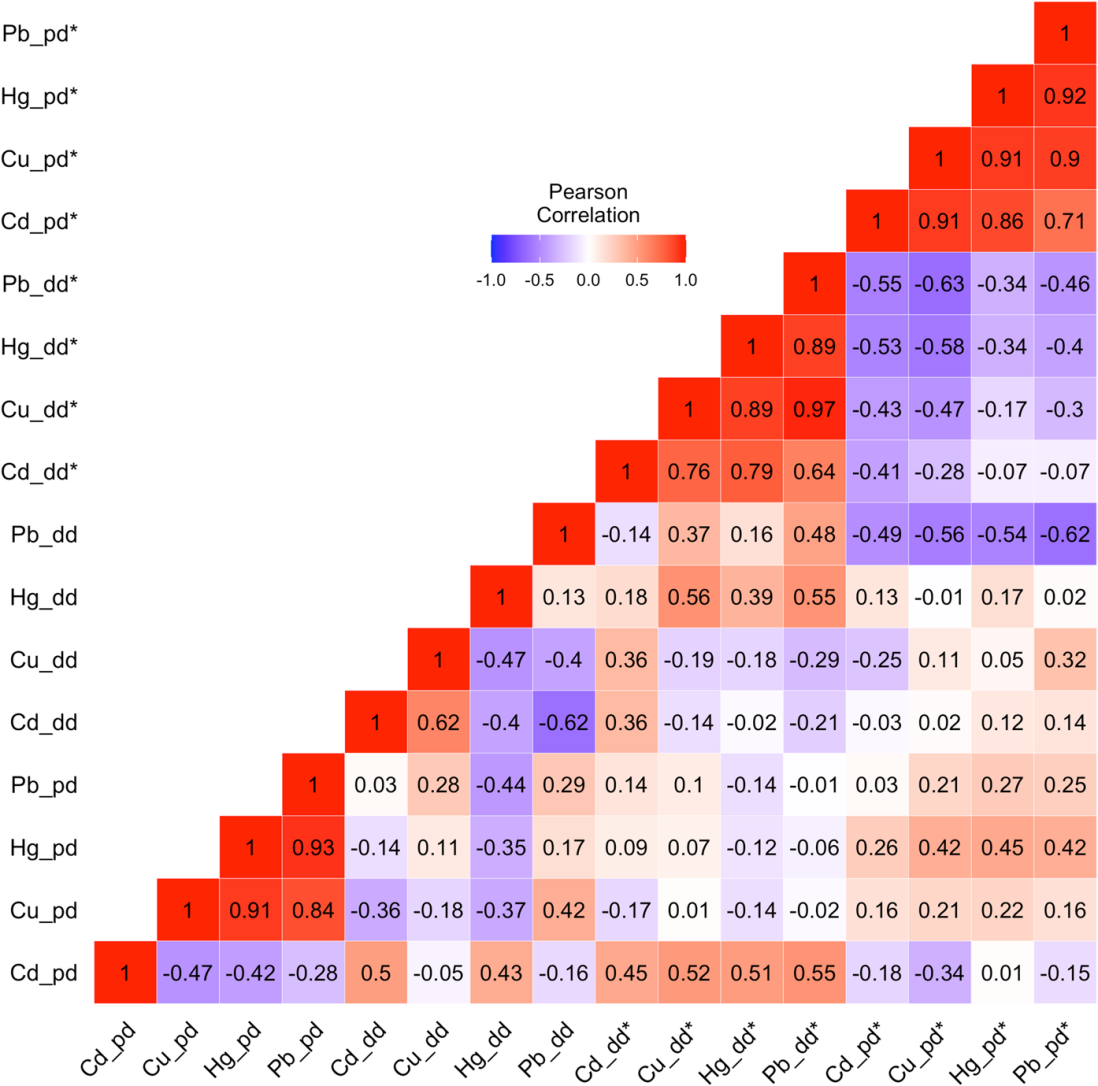
Pearson correlation analysis of trace elements from RM-4 study area across four periods; pd i.e. predredging (August 2011), dd i.e. earlier during dredging (August 2012), dd* i.e. later during dredging (December 2012), and pd* i.e. post-dredging (June 2013)*.* Intercorrelations recorded between August and December 2012 (dd & dd*), indicate potential similarities in sources (majorly resuspension) during the dredging period. Negative intercorrelations between the dredging and post-dredging periods potentially denote re-stabilization and settlement of the river post-dredging, which led to a subsequent reduction in dissolved metal levels

Pearson correlation analysis of toxic metal data for pre-dredging (i.e. August 2011 (pd)), during dredging (i.e. August and December 2012 (dd, dd*)), and post-dredging (i.e. June 2013 (dd*)) revealed strong interrelationships (Fig. 4). Cu_pd_, Hg_pd_, and Pb_pd_ were strongly and significantly correlated (r ≥ 0.84, *p* < 0.01), likewise Cd_dd*_, Cu_dd*_, Hg_dd*_, and Pb_dd*_ were strongly and significantly correlated (r ≥ 0.64, *p* < 0.05), and Cd_pd*_, Cu_pd*_, Hg_pd*_, Pb_pd*_ were all strongly and significantly correlated (r ≥ 0.71, *p*< 0.05). Notably, there was a lack of significant correlation between the metals in August 2012 (dd). Significant negative correlations between dd* and pd* variables denote a potential reduction in water column metal concentrations six months after dredging.

A Kaiser Meyer Olkin score of 0.5 and a significant (*p*<0.05) Bartlett’s sphericity test validated the application of PCA. 2–3 rotated principal components with eigenvalues > 1 and cumulative variances ≥ 82% revealed significant relationships between the metal and physicochemical parameters (Table 2). Strong loading of the metals on PC1 was recorded for August 2011, December 2012, and June 2013. TSS and salinity loaded strongly with the metals in December 2012 and June 2013, respectively. Significant loadings of chlorophyll a on the same components with TSS and salinity were also recorded across the study periods.

**Table 2:** Principal Component Analyses (varimax-rotated component matrix) of physicochemical variables and trace metals in RM-4

August 2011 (pre-dredging)				August 2012 (during dredging)				December 2012 (during dredging)				June 2013 (post-dredging)			
Component	Rotation Sum of Squared Loading			Component	Rotation Sum of Squared Loading			Component	Rotation Sum of Squared Loading			Component	Rotation Sum of Squared Loading		
	Total	% of Variance	% Cum. Variance		Total	% of Variance	% Cum. Variance		Total	% of Variance	% Cum. Variance		Total	% of Variance	% Cum. Variance
1	3.1	45	45	1	2.4	35	35	1	4.4	62	62	1	4.2	60	60
2	2.6	37	82	2	2.1	30	65	2	1.5	22	84	2	2.3	33	93
			3	1.7	25	90									

Variables	Rotated component matrix		Variables	Rotated component matrix			Variables	Rotated component matrix		Variables	Rotated component matrix	
	PC1	PC2		PC1	PC2	PC3		PC1	PC2		PC1	PC2
Hg	0.96	0.1	Pb	0.97	−0.17	−0.08	Cu	0.96	0.17	Hg	0.98	0.044
Cu	0.95	0.07	TSS	0.97	−0.06	−0.14	Hg	0.94	−0.15	Pb	0.94	−0.02
Pb	0.92	0.16	Chl a	0.14	−0.95	−0.06	Pb	0.92	0.02	Cu	0.92	0.36
Chl a	0.25	0.77	Hg	0.05	0.24	−0.87	Cd	0.88	0.09	Cd	0.87	0.41
Sal	0.23	0.84	Sal	−0.01	0.99	−0.1	TSS	0.85	0.4	Sal	0.84	0.41
TSS	−0.08	0.92	Cu	−0.3	0.4	0.78	Chl a	0.43	0.75	Chl a	0.19	0.94
Cd	−0.57	0.62	Cd	−0.67	−0.06	0.56	Sal	0.17	−0.87	TSS	0.13	0.97

### Deterministic and probabilistic health risk assessment

**Fig. 5:**
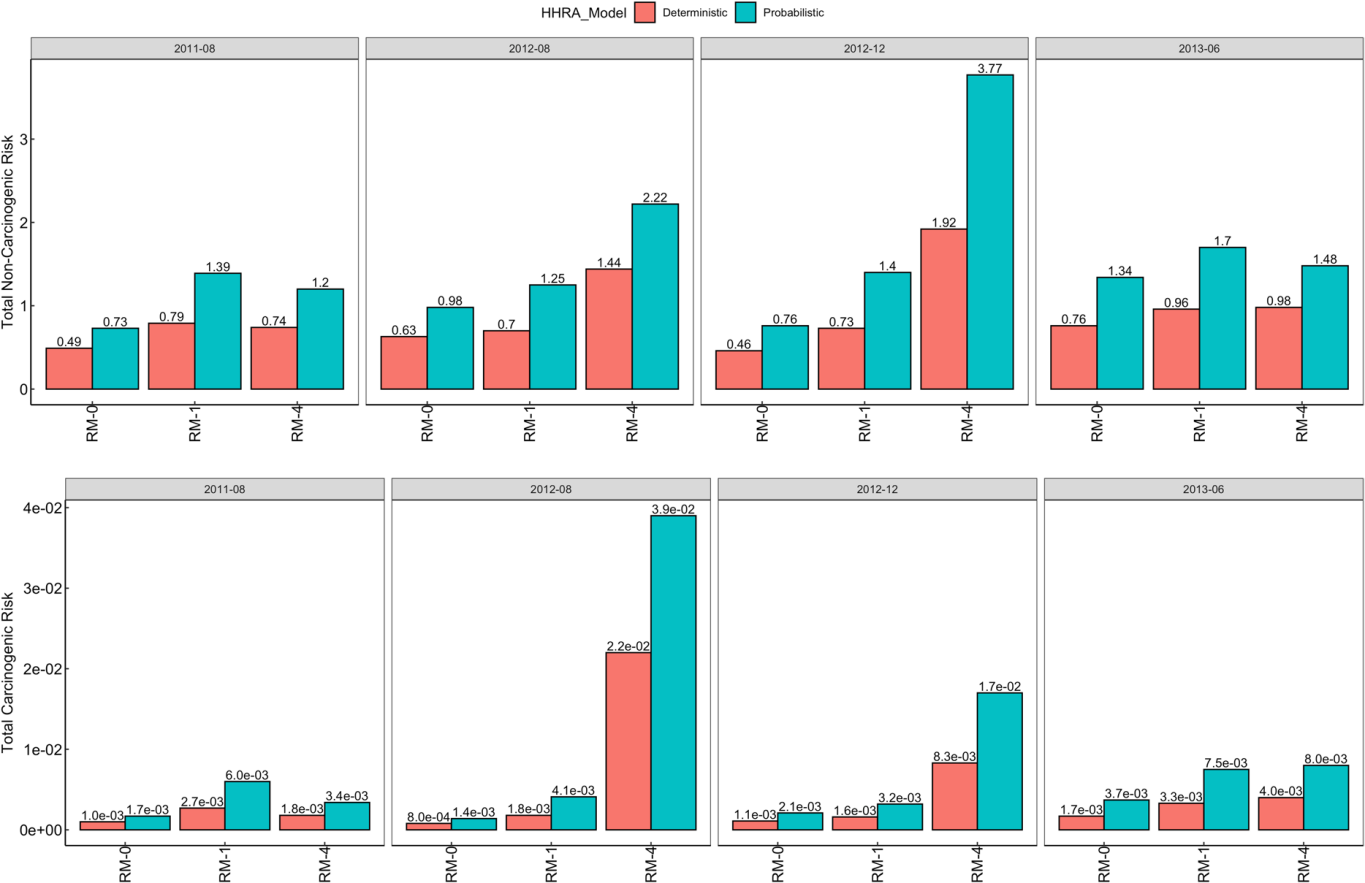
The total carcinogenic (CR) and non-carcinogenic risk (NCR) potential recorded for child population in the study areas, computed using the deterministic and probabilistic HHRA models—The probabilistic model indicated higher risk potential across all periods and study areas (THI > 1, TCR > 10^−4^) while the deterministic model indicated no pre-dredging NCR, although CR was also > 10^−4^ across all periods and study areas. Potential health risks were highest at RM-4, especially during dredging

**Fig. 6:**
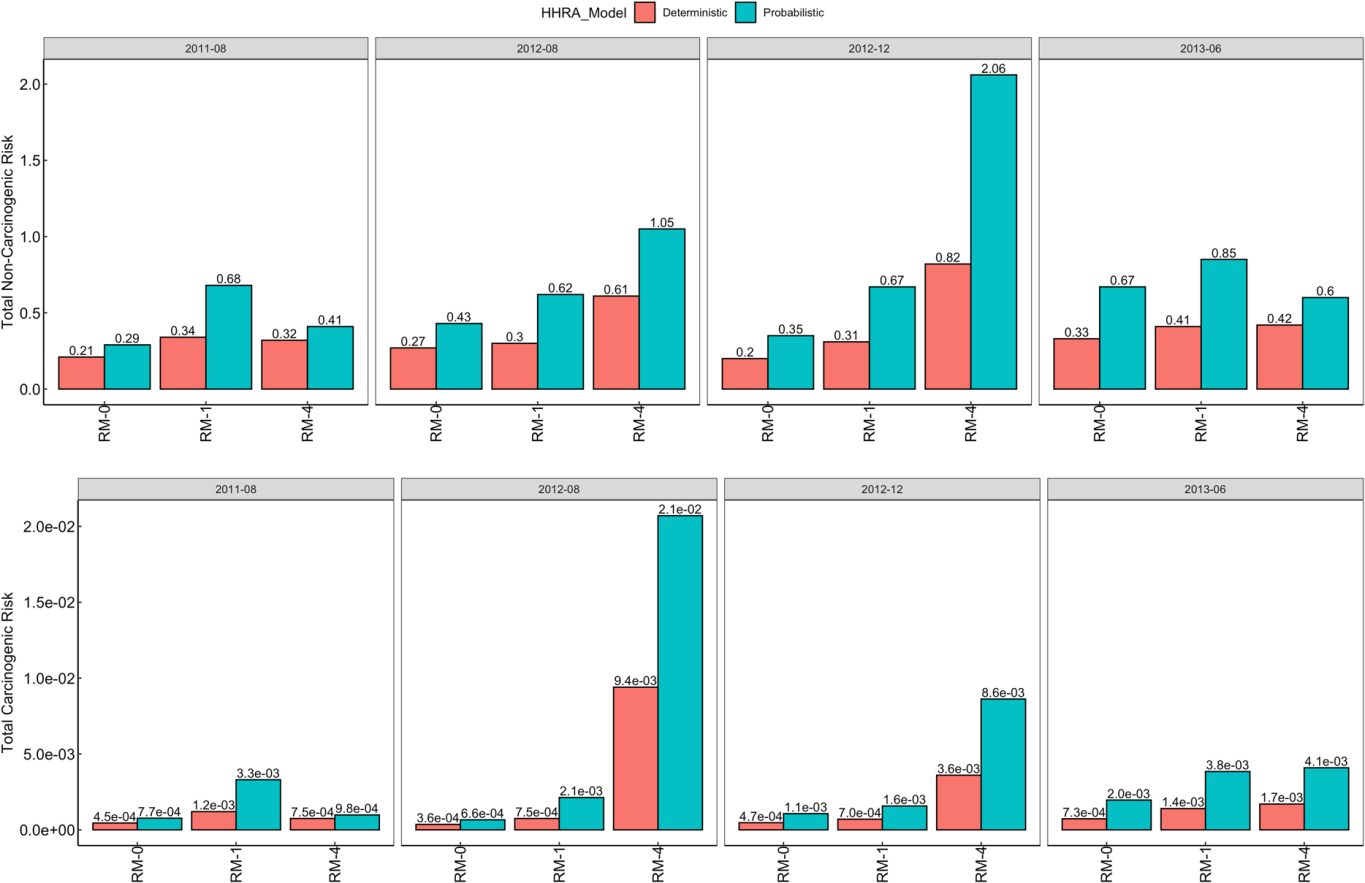
The total carcinogenic (CR) and non-carcinogenic risk (NCR) potential recorded for the adult population in the study areas, as computed using the deterministic and probabilistic HHRA models. The probabilistic model indicated potential NCR for adults exposed only at RM-4 during dredging (August & December 2012). The deterministic model indicated no NCR for adults across all study areas and periods (THI < 1). The deterministic and probabilistic models indicate significant CR for adults across all periods and study areas (TCR > 10^−4^)

The total carcinogenic and non-carcinogenic health risks computed for child and adult populations using the deterministic HHRA model are presented in Figs. 5 and 6, respectively. The deterministic HHRA indicated that non-carcinogenic risk was significant (HI > 1) for children only during dredging (August and December 2012), while adults are at no significant risk of non-carcinogenic effects (HI ≤ 1). Both children and adults are at significant risk of carcinogenic effects (TCR > 10^-4^). The individual metal contributions to health risks are presented in Table S3.

The 95% confidence interval values for the total carcinogenic and non-carcinogenic health risks computed for child and adult populations through the probabilistic HHRA model are also presented in Figs. 5 and 6. These values, together with the 50% and 75% confidence interval values, were obtained by running the data through 10,000 iterations in a Monte Carlo Simulation system (Table S4). The probabilistic HHRA indicated that non-carcinogenic risk was significant for child populations across all periods and study areas, but only for adults exposed in the RM-4 study area during dredging. The probability of significant non-carcinogenic risk was determined as 1–50% in child populations and 0–30% in adult populations (Table S4). Significant risk of carcinogenic effects (TCR > 10^−4^) was recorded in both child and adult populations across all periods and study areas, with the probability of serious carcinogenic risk determined as 98–100%. Sensitivity analysis identified fish ingestion and toxic metals as the most sensitive to health risks (Fig. S3). While Hg was the predominantly sensitive metal to non-carcinogenic risks (≤ 39%), Pb was the most sensitive to carcinogenic risks (≤ 65%).

### Recent status of the study area

**Fig. 7:**
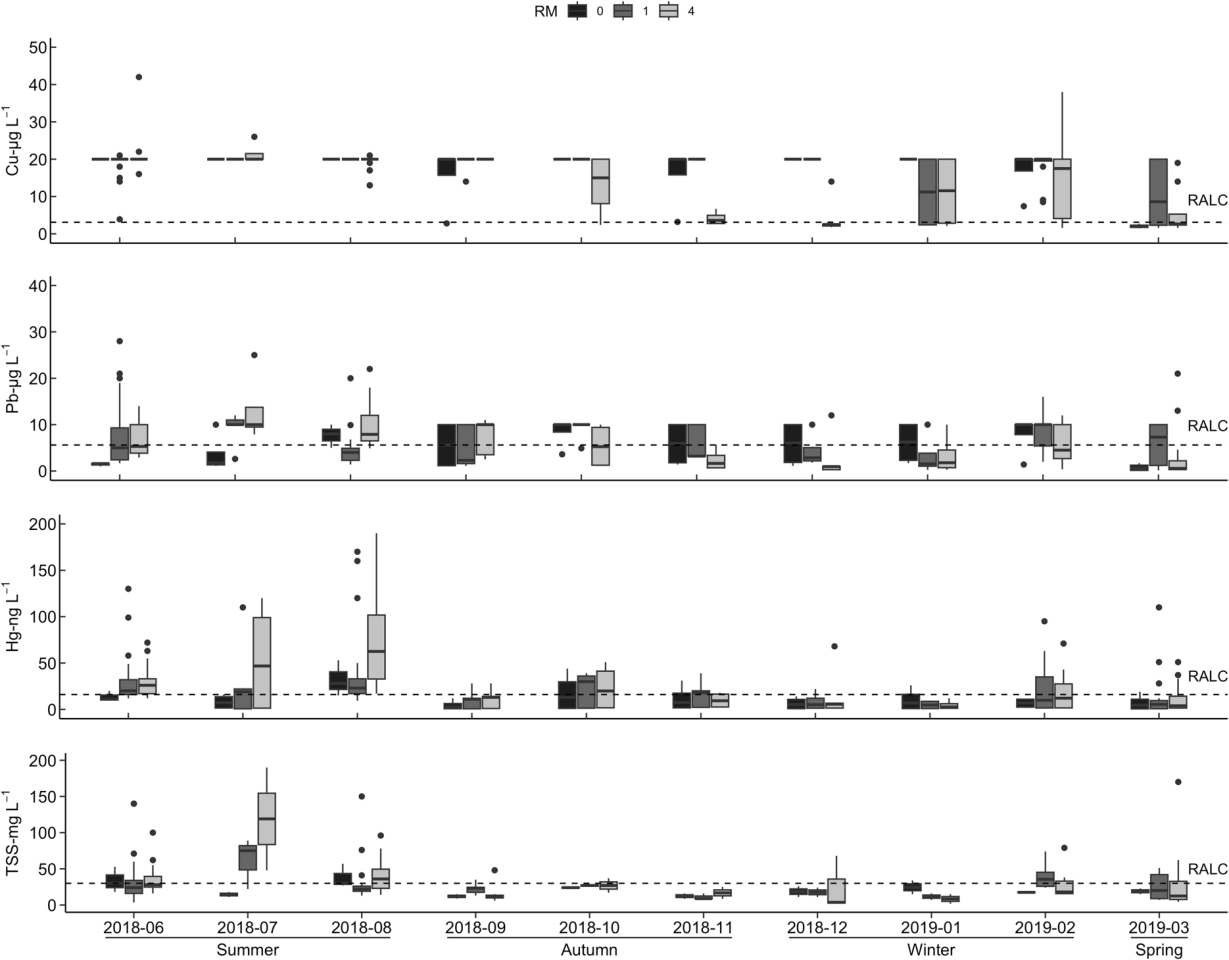
Most recently recorded pollution status of the study areas from June 2018 to March 2019. All the metals recorded varied exceedances of the recommended aquatic life criteria (RALC), but Cu recorded the most consistent and highest exceedance across all periods. The effects of seasons are evident on LPR water quality as measured parameter concentrations changed from Summer to Spring

Analysis of parameter concentrations in LPR water column 6–7 years after the sediment dredging (2018/2019), reveal a significant elevation of metal concentrations from the 2011–2013 levels (Fig. 6). Concentrations of Cu, Hg and Pb in the water column were considerably higher than the criteria levels with recorded increases of 500%, 200% and 130% respectively across all study areas. Consistent with earlier trends, TSS and metal concentrations were higher in the RM-4 study area. Seasonal trends were also detected in the data, with higher TSS and metal concentrations in the summer and autumn months compared to the winter and spring months (Fig. 7).

## Discussion

### Dredging impact on toxic metal pollution and water quality in the LPR

The primary objective of this study was to measure the changes in metal-related pollution and toxicological risks in the LPR water column as a result of dredging operations. While average concentrations of the metals studied were predominantly within safe limits, the maximum metal levels recorded during and post-dredging in the RM-4 sampling locations exceeded the recommended limits by (26–100%), (27–262%) and (5–270%) for Cu, Hg, and Pb respectively, indicating a potentially greater effect of dredging on the study area than RMs-0 and 1. While average metal concentrations may not fully reflect the dire pollution conditions in the LPR, the water quality index reveals a serious threat to aquatic biota in all areas during dredging and in the RM-4 study area across all periods studied. Notably, the worst water quality and metal pollution risks were recorded during and post-dredging, underscoring the effects of RSD on the LPR. Remedial sediment dredging (RSD) disturbs the water-sediment equilibrium, causing oxygen intrusion and pH change (Gustavson et al., 2008). RSD-mediated oxygen ingress into anoxic sediment layers results in anoxic sediment oxygenation, changes in redox and pH conditions, oxidation of metal-sulfide complexes, metal desorption, and displacement from negatively-charged surfaces (Zhang et al., 2014). Furthermore, the mechanical action of dredging releases toxic metals from loosely bound sediment fractions and flocs (Berenjkar et al., 2019), while dissolved metals contained in porewater trapped within the sediment interstices are also released by the squeezing actions associated with dredging (Bridges et al., 2010). Consequently, metal bioavailability and toxicity in the water column are increased. The proximity of the RM-4 study area to the dredging focal point (RM-3.4) denotes that it is likely to be more impacted by the disturbances and geochemical changes associated with dredging. Furthermore, this proximity potentially connotes similar metal levels in RM-4 sediments, which would explain its relatively higher pollution compared to the other study areas. Moreover, dredging residuals, including contaminated sediment plumes and suspended particulate matter (SPM), can travel 3–5 km around the dredged zone and thus, were potentially transported upwards of the dredged area by tidal waves, advection, or dispersion processes (Eggleston, 2012; Fisher et al., 2015; Spearman, 2015).

Metals vary in the way they partition between sediment, suspended particulate matter (SPM), and water, due to their different affinities for sorption to the SPM; this could explain the differences in their dissolved concentrations during dredging (Lebrun et al., 2017; Zhang et al., 2018). Higher enrichment of the SPM by Cd and As compared to other toxic metals during particle-water interactions was reported by Zeng et al. (2019). Also, Monte et al. (2019) applied the bioavailable concentration index to compare metal bioavailability after sediment resuspension. They found that Cd and Pb levels were significantly reduced in the water column. Furthermore, Su et al.,(2022a, 2022b) reported that Cd and Pb can form very stable and insoluble metal-sulfide precipitates when scavenged by free sulfides, while CuS is easily oxidizable during RSD-induced oxygen intrusion.

The role of RSD-induced resuspension as the major cause of organic pollution and metal bioavailability during dredging is highlighted by significant correlations and relationships between metals, chlorophyll-a, and TSS during dredging (Fig. 4, Table 2). Previous reports (Chen et al., 2020, 2021; de Freitas et al., 2019; Moreira et al., 2021) have implicated dredging-related sediment resuspension in aquatic eutrophication and metal intoxication because dredging unearths previously buried sediments contaminated with nutrients and metals, and the disturbance associated with it causes their release from bound forms where they are inactive and non-toxic. Moreover, the significant relationship between salinity and metals in June 2013 (Table 2) potentially points to the role of salinity in trace metal desorption and remobilization from sediments. Previous studies have connected salinity to fluxes in metal concentrations in the sediment and water column (Liu et al., 2019; Zhao et al., 2013). RSD creates a salinity gradient, which causes salt intrusion into the dredged area, thus resulting in the displacement of toxic metals from sediment sorption sites by major cations such as Ca, Mg, K, and Na (Du Laing et al., 2009; Keniston, 2015).

### Ongoing external sources of pollution in the LPR

Notably, poor water quality conditions were recorded, especially in the RM-4 study area before dredging, indicating that RSD is not the root cause of pollution in the LPR. Even after the conclusion of dredging operations in June 2013 or further, in 2018/2019, water quality and metal levels were not significantly better, but rather worse. This confirms anthropogenic involvement and persistence in the LPR. Based on chlorophyll-a concentrations, RM-0 and 1 are categorized as oligotrophic, while RM-4 is mesotrophic (Beiras, 2018). The significant association (p < 0.05) between chlorophyll-a and TSS across all periods in the RM-4 study area has been previously identified as an indicator of nutrient and anthropogenic pollution (Mamun et al., 2022; Sun et al., 2019). Moreover, the tidal status of the LPR denotes that higher TSS levels should be expected in the downstream areas (RMs-0 and 1); however, the distinctly higher TSS levels upstream (RM-4) are indicative of anthropogenic influences from stormwater runoff, combined sewer overflows (CSOs), and municipal waste discharge. CSOs are especially prevalent in the upstream study area, with > 70% of the combined sewer systems in the LPR discharging directly into RMs 4–6 (Nie, 2023). These CSOs comprise stormwater from storm sewers, raw sewage from municipal sewer lines, and permitted industrial effluents, all output into the LPR during storm events to avoid overwhelming the public treatment works (Iannuzzi et al., 1997). Elevated presence of fecal microbes in the LPR has been associated with these CSO outfalls (Donovan et al., 2008). Combined sewer systems are a problematic point source of pollution in the LPR, and their continued usage in New Jersey is bound to continually limit the effectiveness of any remediation attempts on the LPR.

### Seasonal influences on aquatic pollution in the LPR

Most recently available metal and TSS data in the study areas further highlight anthropogenic influences on the LPR. A review of LPR-related studies published in the literature between 2017 and 2019 also alludes to anthropogenic pollution in the river. Jung (2017) and Du et al. (2018) reported that untreated sewage and wastewater discharge into the LPR existed pre-dredging and continued beyond the dredging period. They also reported that the high population density and urbanized shift in land use around the LPR continue to be a major local threat to its ecosystem. Furthermore, the influence that changes in seasons have on the ecological status of the LPR cannot be neglected because seasonal changes influence in situ recontamination dynamics in the LPR. In Fig. 7, TSS and toxic metal concentrations are higher in the summer and autumn compared to the winter and spring. Summer and autumn periods are characterized by heavy rainfall events and flooding, which increase surface runoff intensity, frequency of combined sewer overflows, and resuspension of contaminated sediments, leading to elevated metal levels in the water column (Sheela et al., 2014). Additionally, increased evaporation that characterizes the summer period leads to poor mixing of toxic metals and elevates their concentration in water, while heavy rainfall in the summer and autumn results in indiscriminate wastewater discharge from sewage and public treatment works, mines, landfills, and highways (Chan et al., 2021).

### Health risk assessment and uncertainty

The accuracy of health risk estimation with the probabilistic approach is highlighted in the obtained results. While the deterministic HRA model indicated no risk to adults, i.e., HI < 1, the probabilistic approach revealed the existence of a lower, albeit significant risk probability of non-carcinogenic risk in adults (probability of HI ≥ 1: 0–30%). This difference is important and reflects the robustness of the probabilistic HHRA approach to uncertainties and superior risk characterization which is enabled through the adoption of a range of values for model parameters such as fish ingestion rate, body weight, estimated daily intake, as opposed to the single point value adoption by the deterministic HHRA model (Moe et al., 2022). The elevated non-carcinogenic risk-potential recorded for child and adult populations, especially in the RM-4 study area, reflects the debilitating ongoing and after-effects of RSD on public health and its socio-economic costs, i.e., depriving surrounding communities of safe, unrestricted access to their local aquatic resources for a stipulated length of time. Since humans are at the top of any food chain, the risk of toxic exposure from polluted aquatic systems is grave. Fish is a primary food source for humans and serves as a major source of metal bioaccumulation compared with skin contact and accidental ingestion, with toxic exposure leading to the onset of carcinogenic and non-carcinogenic health complications including cancer, organ damage, skin diseases, immunocompromise, teratogenic effects, vascular damage, neurological defects and gastrointestinal issues (Abalaka et al., 2020; Balali-Mood et al., 2021). Metals such as Cd, Pb, and Hg are non-essential to aquatic organisms and are toxic even at minimal concentrations, impacting an organism’s physiological and metabolic processes. Fish are exposed to these toxicants through trophic transfer, i.e., consumption of smaller fishes and filter-feeding organisms such as zooplankton, which ingest contaminated particulate matter and phytoplankton (Hammerschmidt et al., 2013). They are also exposed through oral ingestion and absorption of polluted water through their gills and skin (Weber et al., 2013). Toxic bioaccumulation is predominant in fish metabolism organs such as the liver, kidney, and gills and is dependent on feeding behavior, swimming methods, sex, and size of the fish, and environmental factors such as metal bioavailability and route of entry (Noman et al., 2022).

## Limitations of the study and future perspectives

This study benefits policymakers and remediation planners as it offers insight into a case study example of sediment dredging impacts on aquatic ecosystems. However, it has a potential limitation that should be addressed in future research. Due to data paucity, only a few water quality indicators, including TSS, alkalinity, and chlorophyll-a, were used for water quality computation. Subsequent environmental impact assessment and monitoring studies on the LPR must employ robust water quality data sampling procedures to conduct comprehensive water quality evaluation using physicochemical indicators, including pH, dissolved oxygen, nutrients (nitrate, orthophosphate, ammonia-nitrogen), and microbiological indicators such as fecal coliform and enterococcus. This is especially crucial to gaining a full understanding of the direct effects of point pollution sources such as CSOs on the LPR, and will thus enable the development of effective source control measures. Furthermore, this research relied significantly on microbial and chemical tests. Future research can complement the results of this study by applying aquatic life toxicological testing measures, including using bioassays and chronic toxicity tests of aquatic organisms (e.g., fish, algae, *Daphnia magnia*, etc.), and whole effluent toxicity (WET) tests to more accurately measure the bioaccumulation of toxicants in aquatic biota of the LPR.

## Conclusion

This environmental assessment of sediment dredging impacts on the LPR system revealed significant toxic metal exceedances of the RALC in the RM-4 study area for Cu (6.5–100%), Hg (25–220%), and Pb (11–24%) during and after dredging. Remedial sediment dredging significantly worsened toxic metal pollution in the LPR water column and degraded water quality.

TSS—metal correlations in the PCA during dredging indicate that RSD was responsible for toxic metal release and resuspension, and dredging residuals were responsible for recontamination. The salinity–metal correlations in the PCA further establish that the salinity gradient caused by RSD led to salinity-induced metal remobilization. Anthropogenic influences—CSOs, stormwater runoff, and industrial wastewaters are the most probable ongoing external pollution sources in the LPR. Along with the changes influenced by seasonality, anthropogenic influences are responsible for the worsened state of toxic metal pollution in the LPR seven years post-dredging. The probabilistic model yielded a more robust health risk assessment, revealing human carcinogenic and non-carcinogenic exposure risk probabilities ranging from 6 to 50% (HI) and > 98% (TCR) in adult and children populations. The fish ingestion route poses the highest exposure risk to humans.

Further remediation work in the LPR must implement stricter source control protocols, contaminant characterization, and pre-remediation risk assessments, and consider a consolidation of remedial technologies to achieve a protective remedy, sustain the effectiveness of the remedy and achieve ecosystem restoration goals and remedial action objectives without doing further damage to the existing biota or surrounding human communities.

## Supplementary Information

Below is the link to the electronic supplementary material.

Supplementary file1 (DOCX 1142 KB)

## Data Availability

All data used/analyzed in this study were obtained from the Passaic River Digital Database** (**sharepoint.ourpassaic.org).
