# Supplementary material for: Spatiotemporal evaluation of water quality, metal pollution, and human health risks in a dredged Urban River, New Jersey, USA
Source: Environ Geochem Health. 2025 Jun 25;47(7):283. doi: 10.1007/s10653-025-02579-6 (PMC12198267; doi:10.1007/s10653-025-02579-6)
Supplement: Supplementary file 1 — Supplementary file1 (DOCX 1142 KB) [file 10653_2025_2579_MOESM1_ESM.docx]

**Supplementary Information**

**Spatiotemporal Evaluation of Water Quality, Metal Pollution, and Human Health Risks in a Dredged Urban Estuarine River, New Jersey, USA**

Oluwafemi Soetan ^a^, Qingzhi Zhu ^b^, Huan Feng ^a^*

^a^ Department of Earth and Environmental Studies, Montclair State University, Montclair, NJ, USA

^b^ School of Marine and Atmospheric Sciences, Stony Brook University, Stony Brook, NY, USA

* Corresponding author: Huan Feng (Email: [fengh@montclair.edu](mailto:fengh@montclair.edu))

Statistical Data Analysis

Data were statistically analyzed for homogeneity and normality using the Bartlett test and Shapiro-Wilks test of normality. Non-normal data were log-transformed before analysis for significant spatiotemporal differences using one-way Analysis of Variance (ANOVA) and Tukey’s HSD post-hoc test. Pearson product-moment correlation coefficient was used to investigate the associations between Cd, Cu, Hg, and Pb data within the same observation period to determine potential similarity in sources. Likewise, the relationship between the metals across the four selected periods (August 2011, August & December 2012, and June 2013) was also investigated to determine potential similarities or changes in metal sources across periods. Principal Component Analysis (PCA) was used to identify the most significant parameters, synthesize inherent information in the data, and thus categorize potential pollution sources. Before principal component analysis, the Kaiser Meyer Olkin (KMO) test of sampling adequacy was used to test for the suitability of the dataset for PCA while the Bartlett test of sphericity was used to investigate the existence of correlations that warrant further analysis. All statistical data analyses and visualization were executed with the R software. Monte Carlo Simulation was used for probabilistic HRA and uncertainty analysis. Oracle Crystal Ball v11.1.3.0 software was used for the Monte Carlo simulation analysis. The spatiotemporal changes in toxic metal concentrations at the most polluted study area (RM-4) were represented using the kriging interpolation method on ArcGIS Pro v 3.0.0.


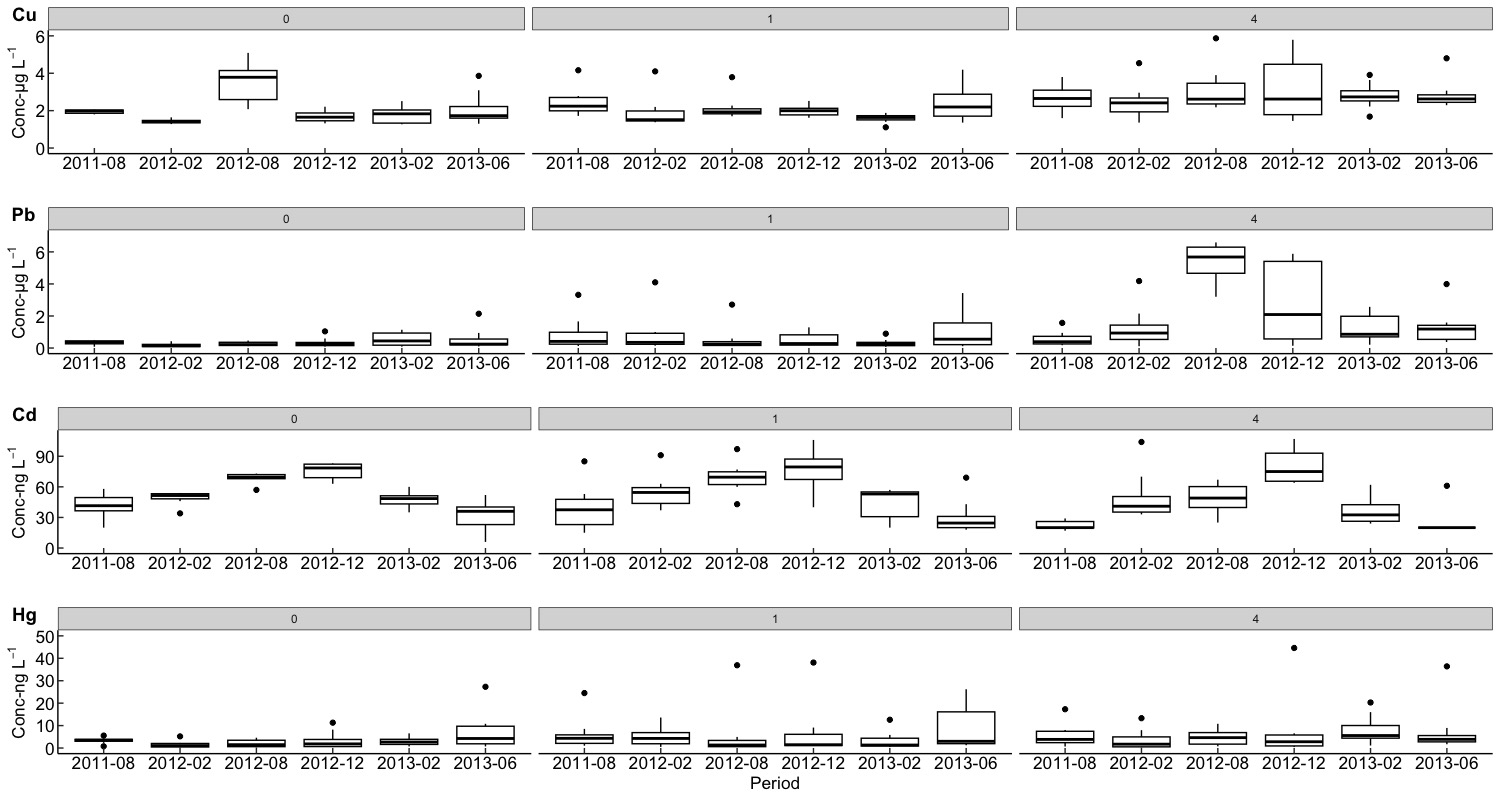


**Figure S1.** Spatiotemporal distribution of trace metal concentrations in the Lower Passaic River study area

**Table S1**.

Parameters used for the water quality index calculation

| Parameter | Chl-a | Cd | Alkalinity | Cu | Hg | TSS | Sulfate | Pb | Salinity | References |
| --- | --- | --- | --- | --- | --- | --- | --- | --- | --- | --- |
| W | 2 | 4 | 3 | 4 | 4 | 4 | 4 | 4 | 1 | (Singh et al., 2020) |
| C_s_ | 20  µg L-1 | 8.8  µg L^-1^ | 120  mg L^-1^ | 3.1  µg L^-1^ | 0.016  µg L^-1^ | 30  mg L^-1^ | 2000  mg L^-1^ | 5.6  µg L^-1^ | 10  g Kg^-1^ | Aquatic Life Criteria |

Reference doses, cancer slope factors, and bioconcentration factors used in HHRA

| Parameter | Cd | Cu | Hg | Pb | References |
| --- | --- | --- | --- | --- | --- |
| RfD_oral_ (µg Kg^-1^d^-1^) | 0.01 | 12 | 0.011 | 0.525 | (Guleria & Chakma, 2021) |
| RfD_dermal_ (µg Kg^-1^d^-1^) | 5 | 40 | 0.16 | 3.5 |  |
| CSF (Kg d mg^-1^) | 6.1 | –– | –– | 8.5 | (Mohammadi et al., 2019) |
| BCF (L Kg^-1^) | 66.45 | 3148.32 | 199.35 | 5000 | (Marty & Blaisdell, 2000; Vu et al., 2017) |


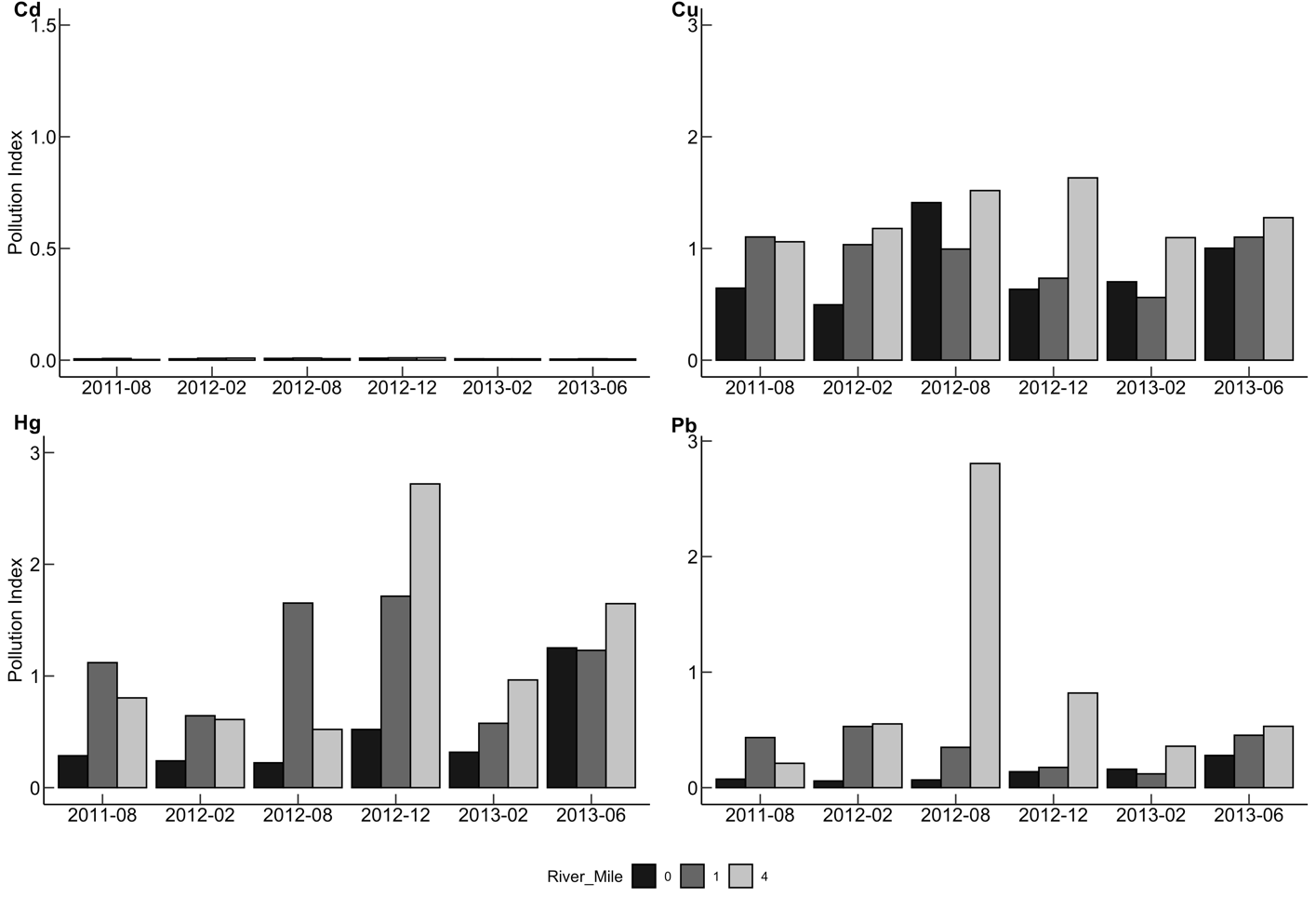


**Figure S2.** The Nemerow pollution index of heavy metals in the study area

**Table S2**. Parameters used for the carcinogenic and non-carcinogenic human health risk assessment (HHRA)

| Parameters | Abbreviation | Child | Adult | Distribution for MCS | References |
| --- | --- | --- | --- | --- | --- |
| Concentration of trace metal | C_n_ (µg L^-1^) | –– | –– | LogNormal | (USEPA, 2004) |
| Coefficient of permeability | PC (cm h^-1^) | 1 x 10^-4^ | 1 x 10^-4^ | Fixed value | (USEPA, 2004) |
| Conversion factor | CF (L cm^-3^) | 1 x 10^-3^ | 1 x 10^-3^ | Fixed value | (USEPA, 2004) |
| Skin contact area | SA (cm^2^) | 6600 | 18000 | Uniform | (Howladar et al., 2021) |
| Exposure time (swimming) | ET_SW_ (h d^-1^) | 1 | 0.58 | Fixed value | (Howladar et al., 2021) |
| Exposure frequency (swimming) | EF_SW_ (d y^-1^) | 350 | 350 | Triangular | (Howladar et al., 2021) |
| Exposure duration (swimming) | ED_sw_ (y) | 6 | 30 | Fixed value | (Howladar et al., 2021) |
| Exposure frequency (fish ingestion) | EF_FI_ (d y^-1^) | 365 | 365 | Triangular | (Vu et al., 2017) |
| Exposure duration (fish ingestion) | ED_FI_ (y) | 6 | 70 | Fixed value | (Vu et al., 2017) |
| Average time | AT (d) | 2100 | 10500 | Fixed value | (Vu et al., 2017) |
| Body weight | BW (Kg) | 15 | 70 | Uniform | (Howladar et al., 2021) |
| Contact rate | R (L h^-1^) | 4.4 x 10^-2^ | 4.4 x 10^-2^ | Fixed value | (Howladar et al., 2021) |
| Average fish ingestion rate | U (Kg d^-1^) | 2.64 x 10^-2^ | 5.28 x 10^-2^ | Triangular | (May & Burger, 1996) |

**Table S3**. Deterministic computation of Target hazard quotient and cancer risk in a) Child b) Adult

|  |  | **Hazard Quotient** | | | | | | | | **Target Cancer Risk** | | | |
| --- | --- | --- | --- | --- | --- | --- | --- | --- | --- | --- | --- | --- | --- |
| **RM 0** | HQ (Child) | | | | | HQ (Adult) | | | | TaCR (Child) | | TaCR (Adult) | |
|  |  | Cd  ( 10^-3^) | Cu | Hg | Pb  ( 10^-1^) | Cd  ( 10^-3^) | Cu | Hg | Pb  ( 10^-1^) | Cd  ( 10^-5^) | Pb (10^-3^) | Cd  ( 10^-5^) | Pb  ( 10^-3^) |
|  | **Aug-11** | 1.14 | 0.27 | 0.18 | 0.34 | 0.41 | 0.12 | 0.08 | 0.15 | 2.93 | 1.01 | 1.26 | 0.43 |
|  | **Feb-12** | 1.37 | 0.20 | 0.09 | 0.18 | 0.49 | 0.08 | 0.04 | 0.08 | 3.51 | 0.54 | 1.50 | 0.23 |
|  | **Aug-12** | 1.91 | 0.49 | 0.11 | 0.26 | 0.69 | 0.21 | 0.05 | 0.11 | 4.90 | 0.78 | 2.10 | 0.34 |
|  | **Dec-12** | 2.11 | 0.23 | 0.19 | 0.35 | 0.76 | 0.10 | 0.08 | 0.15 | 5.41 | 1.04 | 2.32 | 0.44 |
|  | **Feb-13** | 1.32 | 0.25 | 0.16 | 0.56 | 0.48 | 0.11 | 0.07 | 0.24 | 3.39 | 1.66 | 1.45 | 0.71 |
|  | **Jun-13** | 0.89 | 0.29 | 0.41 | 0.57 | 0.32 | 0.12 | 0.18 | 0.24 | 2.29 | 1.68 | 0.98 | 0.72 |
|  |  |  |  |  |  |  |  |  |  |  |  |  |  |
| **RM 1** | **Aug-11** | 1.11 | 0.34 | 0.36 | 0.90 | 0.40 | 0.15 | 0.15 | 0.38 | 2.84 | 2.67 | 1.22 | 1.14 |
|  | **Feb-12** | 1.54 | 0.27 | 0.29 | 0.91 | 0.56 | 0.11 | 0.12 | 0.39 | 3.95 | 2.71 | 1.69 | 1.16 |
|  | **Aug-12** | 1.92 | 0.30 | 0.34 | 0.57 | 0.69 | 0.13 | 0.14 | 0.25 | 4.93 | 1.70 | 2.11 | 0.73 |
|  | **Dec-12** | 2.10 | 0.28 | 0.40 | 0.53 | 0.76 | 0.12 | 0.17 | 0.23 | 5.40 | 1.57 | 2.31 | 0.67 |
|  | **Feb-13** | 1.22 | 0.22 | 0.19 | 0.32 | 0.44 | 0.09 | 0.08 | 0.14 | 3.12 | 0.95 | 1.34 | 0.41 |
|  | **Jun-13** | 0.85 | 0.33 | 0.51 | 1.09 | 0.31 | 0.14 | 0.22 | 0.47 | 2.19 | 3.24 | 0.94 | 1.39 |
|  |  |  |  |  |  |  |  |  |  |  |  |  |  |
| **RM 4** | **Aug-11** | 0.61 | 0.37 | 0.31 | 2.03 | 1.11 | 0.16 | 0.13 | 0.25 | 1.58 | 1.73 | 0.68 | 0.74 |
|  | **Feb-12** | 1.40 | 0.34 | 0.21 | 4.53 | 2.52 | 0.15 | 0.09 | 0.55 | 3.59 | 3.85 | 1.54 | 1.65 |
|  | **Aug-12** | 1.32 | 0.43 | 0.26 | 2.47 | 2.39 | 0.19 | 0.11 | 0.30 | 3.41 | 2.10 | 1.46 | 0.90 |
|  | **Dec-12** | 2.22 | 0.50 | 1.15 | 9.66 | 4.02 | 0.21 | 0.49 | 1.18 | 5.72 | 8.21 | 2.45 | 3.52 |
|  | **Feb-13** | 1.01 | 0.39 | 0.44 | 4.28 | 1.83 | 0.17 | 0.19 | 0.52 | 2.61 | 3.64 | 1.12 | 1.56 |
|  | **Jun-13** | 0.70 | 0.40 | 0.44 | 4.70 | 1.26 | 0.17 | 0.19 | 0.58 | 1.79 | 3.99 | 0.77 | 1.71 |


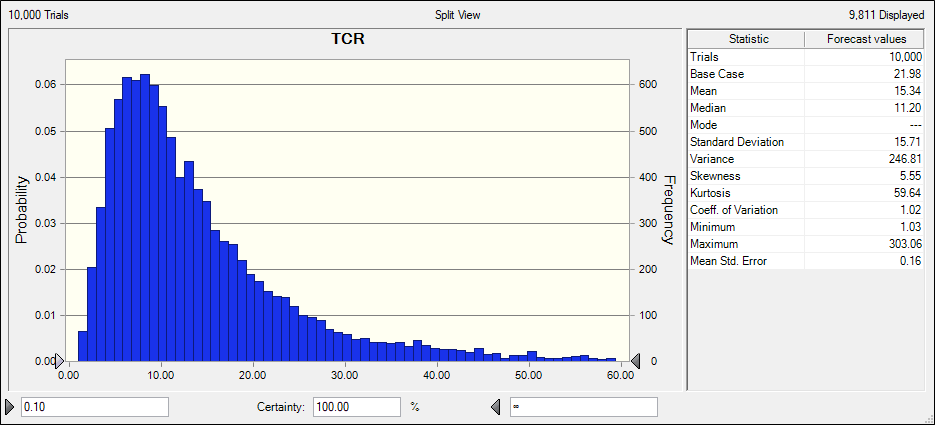

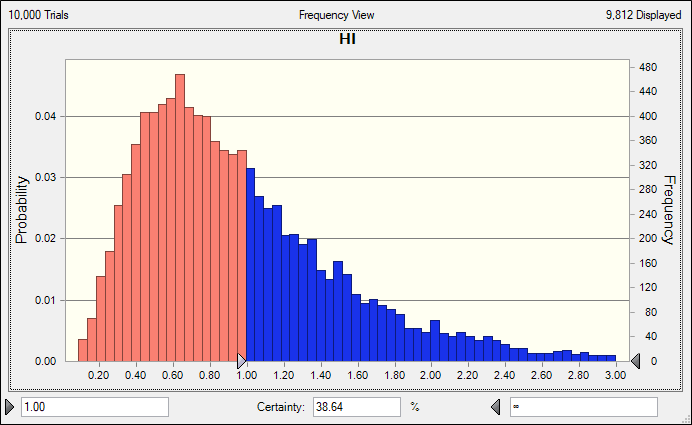

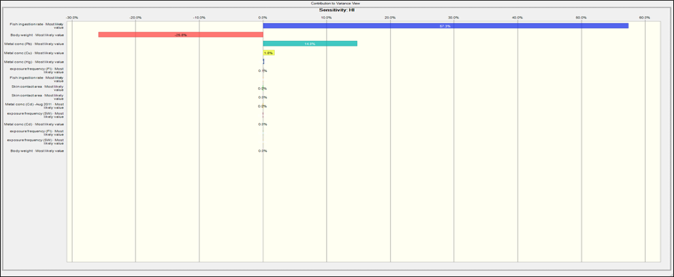

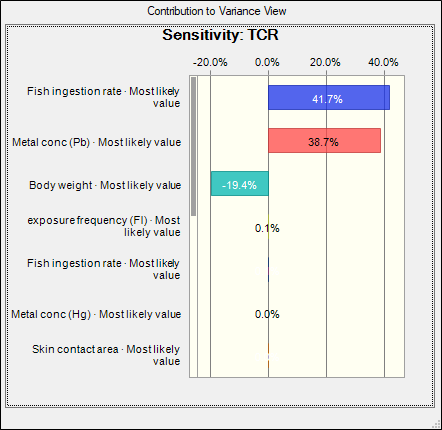


a


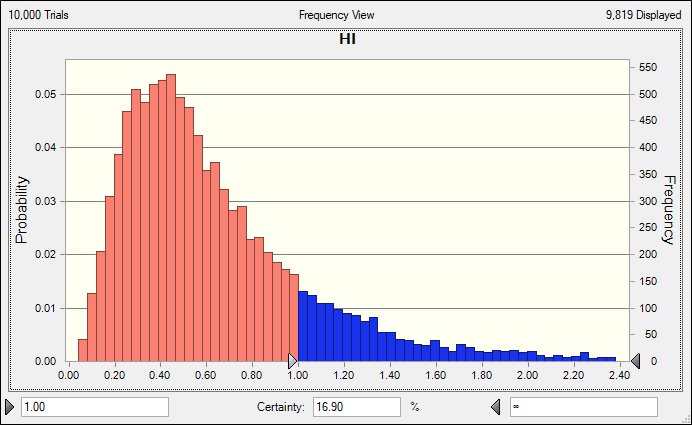

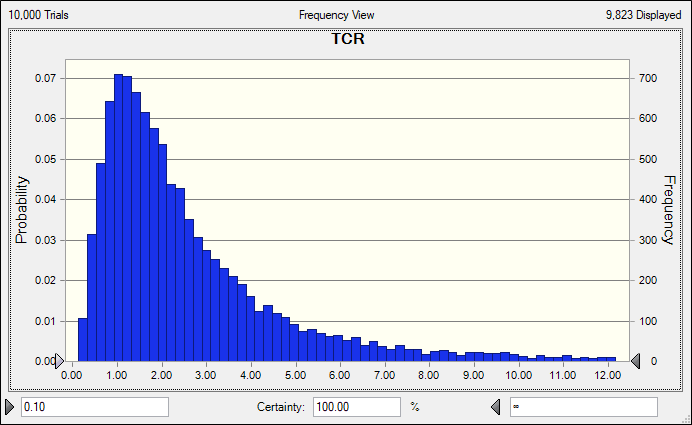

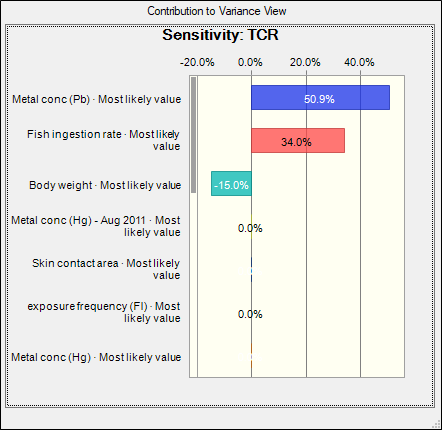

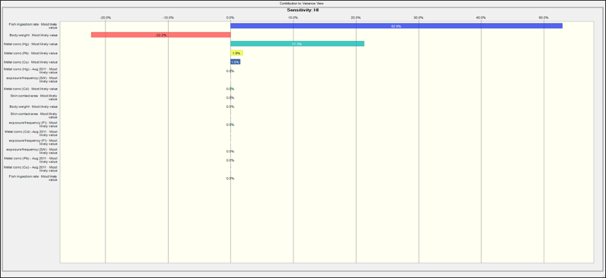


b


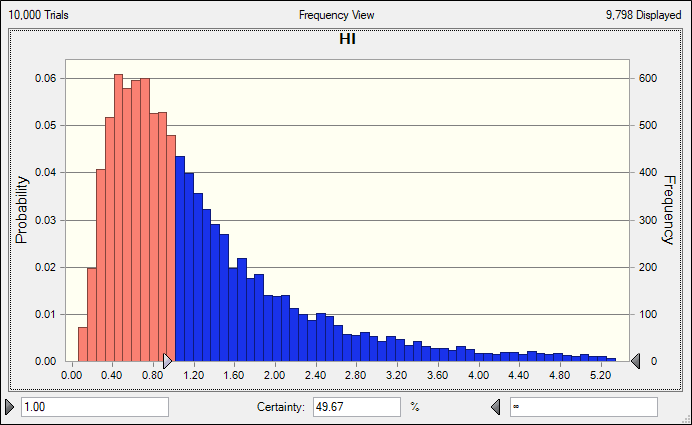

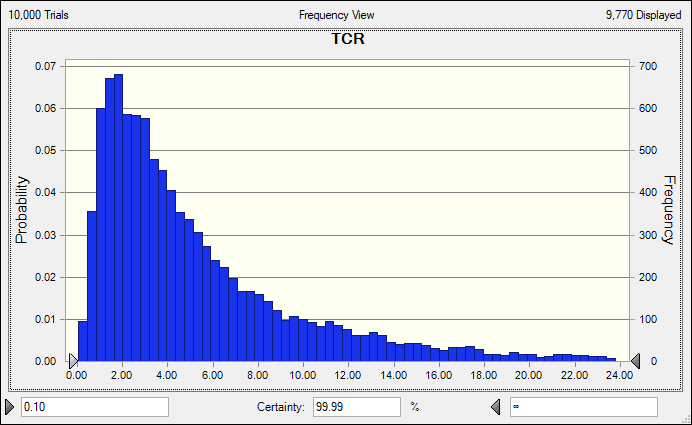

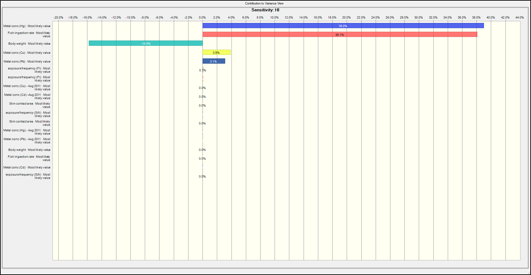

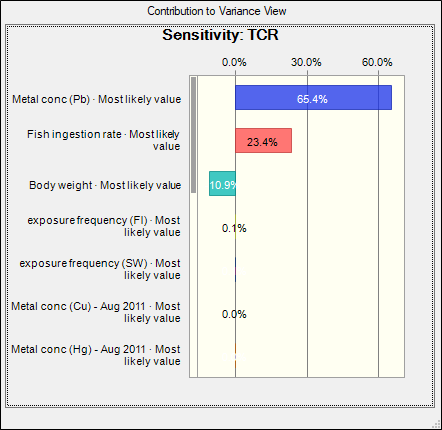


c

**Figure S3.** a) August 2012 RM4 Child HI and TCR Histogram and Sensitivity chart, b) December 2012 RM4 Child HI and TCR Histogram and Sensitivity chart, c) June 2013 RM4 Child HI and TCR Histogram and Sensitivity chart

**Table S4** Carcinogenic and Non-carcinogenic risk determined by probabilistic approach Monte Carlo

|  | Population | Probability | Aug-11 | Aug-12 | Dec-12 | Jun-13 |  | Probability | Aug-11 | Aug-12 | Dec-12 | Jun-13 |
| --- | --- | --- | --- | --- | --- | --- | --- | --- | --- | --- | --- | --- |
|  | **RM-0** |  |  |  |  |  |  |  |  |  |  |  |
| **HI** | Child | 0.5 | 0.31 | 0.39 | 0.27 | 0.41 | **TCR(10^-3^)** | 0.5 | 0.62 | 0.47 | 0.54 | 0.71 |
|  |  | 0.75 | 0.45 | 0.57 | 0.41 | 0.66 |  | 0.75 | 0.95 | 0.73 | 0.93 | 1.35 |
|  |  | 0.95 | 0.73 | 0.98 | 0.76 | 1.34 |  | 0.95 | 1.71 | 1.37 | 2.14 | 3.73 |
|  |  | Mean | 0.35 | 0.45 | 0.33 | 0.54 |  | Mean | 0.75 | 0.58 | 0.78 | 1.2 |
|  |  | Prob > 1 (%) | 0.85 | 4.44 | 2.09 | 10.54 |  | Prob >  1 ✕ 10^-4^ (%) | 99.6 | 98.9 | 98.8 | 99.3 |
|  | Adult | 0.5 | 0.19 | 0.23 | 0.16 | 0.25 |  | 0.5 | 0.37 | 0.28 | 0.31 | 0.41 |
|  |  | 0.75 | 0.23 | 0.3 | 0.21 | 0.35 |  | 0.75 | 0.5 | 0.38 | 0.5 | 0.75 |
|  |  | 0.95 | 0.29 | 0.43 | 0.35 | 0.67 |  | 0.95 | 0.77 | 0.66 | 1.07 | 1.96 |
|  |  | Mean | 0.19 | 0.25 | 0.18 | 0.3 |  | Mean | 0.41 | 0.32 | 0.43 | 0.67 |
|  |  | Prob > 1 (%) | 0 | 0.03 | 0.14 | 1.54 |  | Prob >  1 ✕ 10^-4^ (%) | 99.6 | 98.3 | 97.8 | 98.6 |
|  | **RM-1** |  |  |  |  |  |  |  |  |  |  |  |
|  | Child | 0.5 | 0.46 | 0.35 | 0.38 | 0.52 |  | 0.5 | 1.13 | 0.7 | 0.82 | 1.39 |
|  |  | 0.75 | 0.71 | 0.56 | 0.6 | 0.83 |  | 0.75 | 2.17 | 1.33 | 1.39 | 2.67 |
|  |  | 0.95 | 1.39 | 1.25 | 1.4 | 1.7 |  | 0.95 | 5.95 | 4.08 | 3.19 | 7.45 |
|  |  | Mean | 0.57 | 0.49 | 0.52 | 0.68 |  | Mean | 1.28 | 1.27 | 1.18 | 2.34 |
|  |  | Prob > 1 (%) | 12.2 | 7.94 | 9.44 | 17.73 |  | Prob >  1 ✕ 10^-4^ (%) | 99.8 | 99.2 | 99.8 | 99.9 |
|  | Adult | 0.5 | 0..27 | 0.2 | 0.22 | 0.31 |  | 0.5 | 0.67 | 0.4 | 0.48 | 0.84 |
|  |  | 0.75 | 0.38 | 0.29 | 0.31 | 0.45 |  | 0.75 | 1.24 | 0.72 | 0.75 | 1.53 |
|  |  | 0.95 | 0.68 | 0.62 | 0.67 | 0.85 |  | 0.95 | 3.3 | 2.13 | 1.57 | 3.84 |
|  |  | Mean | 0.31 | 0.28 | 0.29 | 0.38 |  | Mean | 0.68 | 0.68 | 0.64 | 1.31 |
|  |  | Prob > 1 (%) | 1.4 | 2.01 | 2.15 | 3.06 |  | Prob >  1 ✕ 10^-4^ (%) | 99.7 | 98.8 | 99.8 | 99.8 |
|  | **RM-4** |  |  |  |  |  |  |  |  |  |  |  |
|  | Child | 0.5 | 0.43 | 0.84 | 0.99 | 0.61 |  | 0.5 | 0.88 | 12 | 3.89 | 1.97 |
|  |  | 0.75 | 0.66 | 1.26 | 1.67 | 0.88 |  | 0.75 | 1.48 | 18.2 | 7.27 | 3.37 |
|  |  | 0.95 | 1.2 | 2.22 | 3.77 | 1.48 |  | 0.95 | 3.37 | 39.3 | 17.3 | 8.01 |
|  |  | Mean | 0.54 | 1.02 | 1.38 | 0.7 |  | Mean | 1.24 | 13.2 | 5.85 | 2.93 |
|  |  | Prob > 1 (%) | 8.73 | 38.64 | 49.67 | 16.9 |  | Prob >  1 ✕ 10^-4^ (%) | 99.8 | 100 | 100 | 100 |
|  | Adult | 0.5 | 0.3 | 0.52 | 0.76 | 0.38 |  | 0.5 | 0.7 | 6.72 | 2.33 | 1.17 |
|  |  | 0.75 | 0.35 | 0.66 | 1.08 | 0.46 |  | 0.75 | 0.82 | 9.87 | 4.02 | 1.84 |
|  |  | 0.95 | 0.41 | 1.05 | 2.06 | 0.6 |  | 0.95 | 0.98 | 20.7 | 8.62 | 4.09 |
|  |  | Mean | 0.3 | 0.56 | 0.77 | 0.38 |  | Mean | 0.68 | 8.5 | 3.23 | 1.59 |
|  |  | Prob > 1 (%) | 0.5 | 6.13 | 29.5 | 0.14 |  | Prob >  1 ✕ 10^-4^ (%) | 99.8 | 100 | 100 | 100 |

**References**

Howladar, M. F., Hossain, M. N., Anju, K. A., & Das, D. (2021). Ecological and health risk assessment of trace metals in water collected from Haripur gas blowout area of Bangladesh. *Scientific Reports*, *11*(1), 15573. https://doi.org/10.1038/s41598-021-94830-0

May, H., & Burger, J. (1996). Fishing in a polluted estuary: Fishing behavior, fish consumption, and potential risk. *Risk Analysis*, *16*(4), 459–471. https://doi.org/10.1111/j.1539-6924.1996.tb01093.x

Singh, A. K., Sathya, M., Verma, S., & Jayakumar, S. (2020). Spatiotemporal variation of water quality index in Kanwar wetland, Begusarai, India. *Sustainable Water Resources Management*, *6*(3), 1–8. https://doi.org/10.1007/s40899-020-00401-y

USEPA. (2004). *Risk Assessment Guidance for Superfund Volume I: Human Health Evaluation Manual (Part E, Supplemental Guidance for Dermal Risk Assessment) ABOUT THIS DOCUMENT* (Issue July).

Vu, C. T., Lin, C., Yeh, G., & Villanueva, M. C. (2017). Bioaccumulation and potential sources of heavy metal contamination in fish species in Taiwan: assessment and possible human health implications. *Environmental Science and Pollution Research*, *24*(23), 19422–19434. https://doi.org/10.1007/s11356-017-9590-4
